# Mast Cell–Microglia Crosstalk Drives Aberrant Synaptic Pruning Through PAR‐2/MAPK/NF‐κB Signaling in Neonatal Hypoxic–Ischemic Encephalopathy

**DOI:** 10.1002/cns.71189

**Published:** 2026-09-28

**Authors:** Yanping Yang, Dingliang Cai, Jingwen Yu, Yixiao Zhu, Yongxin Huang, Yubing Lin, Chengqian Teng, Jianjie Wei, Ying Lin, Dongsheng Dai, Fushan Xue, Andi Chen, Xiaochun Zheng, Xiaohui Chen

**Affiliations:** ^1^ Shengli Clinical Medical College of Fujian Medical University, Department of Anesthesiology Fujian Provincial Hospital, Fuzhou University Affiliated Provincial Hospital Fuzhou Fujian China; ^2^ Fujian University of Traditional Chinese Medicine, Department of Anesthesiology Fujian Provincial Hospital, Fuzhou University Affiliated Provincial Hospital Fuzhou Fujian China

**Keywords:** hypoxic–ischemic, mast cell, synaptic pruning, tryptase

## Abstract

**Aims:**

Neonatal hypoxic–ischemic encephalopathy (HIE) causes severe neurodevelopmental impairment, but the upstream immune mechanisms that initiate this process remain unclear. We investigated whether mast cell (MC)‐derived tryptase contributes to aberrant microglia‐mediated synaptic pruning through PAR‐2/MAPK/NF‐κB signaling after neonatal hypoxic–ischemic (HI) injury.

**Methods:**

A postnatal Day‐7 rat HI model was established using the Rice‐Vannucci method. MC abundance, c‐Kit and tryptase expression, PAR‐2/MAPK/NF‐κB signaling, and synaptic integrity were assessed using histological, immunofluorescence, biochemical, ultrastructural, and three‐dimensional (3D) reconstruction analyses. Microglia‐targeted F2rl1 silencing and pharmacological inhibition with FSLLRY‐NH2 or APC366 were used to examine PAR‐2 and tryptase‐related signaling. Complementary oxygen–glucose deprivation/reperfusion (OGD/R) experiments were performed in BV‐2 microglia. Cognitive outcomes were assessed using the Morris water maze and Y maze.

**Results:**

HI increased hippocampal MC abundance and tryptase expression, and these changes were associated with acute neurological deficits. HI insult also increased complement associated synaptic labeling, PAR‐2/MAPK/NF‐κB signaling, CD68 expression, and engulfment of PSD95 positive synaptic material. Microglia‐targeted F2rl1 silencing attenuated pathway activation and synaptic engulfment. FSLLRY‐NH2 and APC366 similarly reduced HI associated signaling and microglial synaptic engulfment, while APC366 preserved dendritic spine density and synaptic ultrastructure and improved long‐term spatial learning and memory. In BV‐2 microglial cells, exogenous tryptase enhanced OGD/R associated CD68 expression and MAPK/NF‐κB phosphorylation, which were attenuated by F‐NH2.

**Conclusion:**

MC‐derived tryptase is an upstream contributor to pathological microglial synaptic pruning and cognitive impairment after neonatal HI, potentially involving the PAR‐2/MAPK/NF‐κB axis.

## Graphical abstract

Following neonatal hypoxic–ischemic injury, hippocampal mast cell degranulation releases tryptase, which activates microglial PAR‐2 receptors to trigger p38 MAPK/NF‐κB signaling and drive aberrant synaptic engulfment. This pathological mast cell–microglia crosstalk leads to progressive synaptic loss and long‐term cognitive impairment. Pharmacological blockade of tryptase or PAR‐2 suppresses aberrant microglial pruning activity, preserves synaptic integrity, and rescues spatial memory deficits.

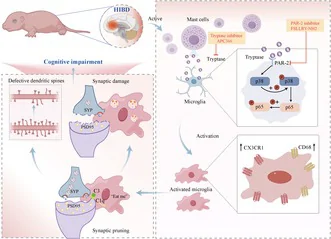

## 1 Introduction

Neonatal hypoxic–ischemic encephalopathy (HIE) is a brain injury disease resulting from the disruption of cerebral blood flow and oxygen supply during birth. It is one of the most common acquired brain injuries in newborns and remains a leading cause of long‐term neurodevelopmental impairment [1]. However, the cellular and molecular mechanisms underlying neural injury in HIE have not been fully elucidated. Most previous research has primarily focused on the interactions among three types of brain cells: activated microglia, reactive astrocytes, and injured or apoptotic neurons [2, 3, 4]. Although potential mechanisms underlying how glial cells contribute to neuroinflammatory cascades and neuronal damage have been identified, the upstream role of other immune cell populations in regulating microglial activation and subsequent neuronal injury remains poorly understood and warrants further investigation.

MC are multifunctional immune cells that play a pivotal role in regulating and monitoring the body's inflammatory responses [5, 6]. In the central nervous system (CNS), they act as early responders to brain injury and can trigger neuroinflammatory cascades before resident glial cells are activated [7, 8]. MC activation has been implicated in several neurological disorders and is thought to amplify neuroinflammation and secondary neuronal damage through interactions with glial and vascular compartments [9, 10, 11, 12, 13]. Previous studies have shown that MC activation occurs at an early stage after HI and is accompanied by increased MC number and inflammatory mediator release in rats. Recent evidence further suggests that hippocampal MC may contribute to BBB permeability and immune cell infiltration after brain injury in mice [13, 14]. However, it remains unclear whether mast cell activation interacts with microglia and thereby contributes to the initiation and progression of brain injury in HIE.

Evidence suggests that MC‐derived mediators, including histamine, serotonin, and tryptase, can modulate microglial function, suggesting that microglia may represent an important downstream effector of MC signaling [8, 15]. Microglia are key regulators of synaptic pruning during CNS development and selectively engulf redundant synapses through molecular cues such as complement proteins C3 and C1q [16, 17]. The fractalkine receptor C‐X3‐C motif chemokine receptor 1 (CX3CR1) contributes to microglia–neuron communication, adhesion, and chemotaxis, whereas CD68 is associated with lysosomal and phagocytic activity [18, 19]. Under pathological conditions, dysregulated microglia‐mediated synaptic pruning can lead to excessive synapse loss and neurological dysfunction [20, 21]. However, it remains unclear whether HI disrupts microglia‐mediated synaptic pruning during the early postnatal period, a critical developmental window when this process is highly active. In addition, the upstream molecular signals and cellular mediators that drive the shift from physiological to pathological synaptic pruning have yet to be fully elucidated.

Recent studies have demonstrated that tryptase derived from MC can cleave and activate protease‐activated receptor‐2 (PAR‐2) on microglia, which may trigger mitogen‐activated protein kinase (MAPK) phosphorylation and modulate microglial function through the NF‐κB signaling pathway [8, 22, 23]. These findings suggest that PAR‐2 may contribute to MC‐microglia communication. Consistent with this possibility, PAR‐2 expression is upregulated in microglia in both the HI model and the oxygen–glucose deprivation/reperfusion (OGD/R) model, suggesting its potential involvement in HIE pathophysiology [24]. Additional evidence from other neuroinflammatory models identifies PAR‐2 as an important regulator of glia‐driven CNS pathology [15]. However, whether MC‐derived tryptase engages microglial PAR‐2 to excessive synaptic pruning via the PAR‐2/MAPK/NF‐κB axis in neonatal rats remains to be determined.

We hypothesized that HI insult‐induced tryptase activates microglial PAR‐2/MAPK/NF‐κB signaling and promotes synaptic damage and cognitive dysfunction through excessive PSD95 engulfment.

## 2 Materials and Methods

### 2.1 Animals and Experimental Design

All experimental procedures were performed using postnatal Day 7 (P7) male and female Sprague–Dawley rats subjected to the Rice–Vannucci hypoxic–ischemic (HI) model. Animals were randomized within litters, and all feasible analyses were conducted under coded, blinded conditions to minimize experimental bias. Detailed descriptions of the experimental design, group allocation, and ethical protocols are provided in Supporting Informations and Methods 1.1.

### 2.2 Histological Staining and 2,3,5‐Triphenyltetrazolium Chloride (TTC) Staining

Cerebral injury and mast cell‐related signaling alterations were evaluated via multiple staining approaches, including H&E, TTC, Nissl, and TUNEL staining, as well as c‐Kit/tryptase dual labeling. Detailed operational procedures are specified in Supporting Informations and Methods 1.2 and Table S1.

### 2.3 RNA Sequencing and Library Construction

Bulk RNA sequencing (RNA‐seq) of hippocampal tissues was performed following HI injury, with full methodological details provided in Supporting Informations and Methods 1.3.

### 2.4 Behavioral Assessments of Acute Neurological Impairment

Acute neurological dysfunction was evaluated at 24 h post‐HI injury. Relevant experimental methods are documented in Supporting Informations and Methods 1.4.

### 2.5 Intranasal Drug Administration

F‐NH2 (a selective PAR2 antagonist) or APC366 (a specific mast cell tryptase inhibitor) was delivered via intranasal administration after HI surgery or sham operation, with matched vehicle groups set as controls. Detailed administration protocols are available in Supporting Informations and Methods 1.5.

### 2.6 Microglia‐Targeted F2rl1 Silencing

For microglia‐targeted F2rl1 knockdown, AAV9‐CX3CR1‐shF2rl1 or control shRNA was stereotaxically injected into the rat hippocampus on P1 prior to HI injury. Detailed procedures are described in Supporting Informations and Methods1.6 [25, 26].

### 2.7 BV‐2 Cell OGD/R and Pharmacological Treatments

BV‐2 microglial cells were subjected to oxygen–glucose deprivation/reoxygenation (OGD/R) injury, followed by treatment with vehicle, F‐NH2, tryptase, or their combination. Detailed cell experiments and pharmacological intervention protocols are provided in Supporting Informations and Methods 1.7.

### 2.8 Western Blotting

Western blotting was performed to detect the expression of target proteins. Protein expression was quantified with β‐actin as the internal reference, and phosphorylated protein levels were normalized to their respective total protein levels. Detailed experimental procedures are listed in Supporting Informations and Methods 1.8 and Table S1.

### 2.9 Immunofluorescence Staining

Immunofluorescence staining, single‐channel quantitative analysis, ImageJ‐based surface visualization, and Z‐stack three‐dimensional (3D) reconstruction were conducted as previously described in Supporting Informations and Methods 1.9 and Table S1.

### 2.10 Transmission Electron Microscopy

Transmission electron microscopy (TEM) was utilized to observe and analyze the ultrastructural characteristics of hippocampal synapses. Relevant methods are provided in Supporting Informations and Methods 1.10.

### 2.11 Golgi Staining

Golgi staining was applied to assess neuronal dendritic morphology and spine density in hippocampal tissues. Detailed protocols are shown in Supporting Informations and Methods 1.11.

### 2.12 Behavioral Tests of Cognitive Function

Cognitive function was evaluated via Y‐maze and Morris water maze tests starting at P30 (23 days after HI injury). Specific testing procedures are described in Supporting Informations and Methods 1.12.

### 2.13 Statistical Analysis

Statistical analyses were performed using SPSS 22.0 software (SPSS Inc., USA). Normality was assessed using the Shapiro–Wilk test, and homogeneity of variance was evaluated using Levene's test. Normally distributed data are presented as mean ± standard error of the mean (SEM). A *p* value < 0.05 was considered statistically significant. Detailed statistical methods are provided in Supporting Informations and Methods 1.13.

## 3 Results

### 3.1 Mast Cell Activation and Tryptase Expression Were Associated With Early Behavioral Deficits After HI Insult

We first validated successful establishment of the Rice‐Vannucci neonatal HI model. TTC staining demonstrated prominent infarction within the ipsilateral hemisphere in HI injured animals relative to sham controls (Figure S1A,B). TUNEL staining revealed enhanced neuronal apoptosis, and Nissl staining showed evident neuronal loss in the hippocampus (Figure S1C–F). We then investigated hippocampal MC activation and tryptase production after HI. Compared with sham rats, HI animals exhibited expanded c‐Kit‐ and tryptase‐positive signals, greater numbers of morphologically identifiable mast cells, and increased cKit^+^/tryptase^+^ double‐positive cells in the hippocampus (Figure 1A–F). Western blotting further confirmed sustained upregulation of tryptase within 24 h post‐HI (Figure 1G,H). At 24 h after injury, HI rats displayed acute neurological impairment, reflected by prolonged latencies for the righting‐reflex, cliff‐avoidance, and negative‐geotaxis tests, alongside shortened forelimb‐suspension duration (Figure 1I–L). Correlation analyses demonstrated that hippocampal mast‐cell abundance and tryptase levels positively correlated with these response latencies and negatively correlated with forelimb‐suspension time (Figure S2), linking mast‐cell activation and tryptase expression to early neurological dysfunction. To dissect the downstream molecular signatures accompanying mast‐cell‐driven pathology, we performed bulk RNA‐seq on post‐HI hippocampal tissue. Volcano‐plot analysis identified 208 significantly up‐regulated and 180 down‐regulated genes in HI versus sham rats (Figure 1M). A targeted heatmap illustrated coordinated transcriptional alterations, including complement‐related genes (*C1qa*, *C1qb*, *C1qc*, *C3*), mast‐cell‐associated proteases and receptors (*Cma1*, *Tpsab1*, *Tpsb2*, *Cpa3*, *Kit*, *Fcer1g*), pro‐inflammatory mediators (*Il1b*, *Il6*), the “don't‐eat‐me” signal *Cd47*, phagolysosomal activity, and microglia–neuron communication related transcripts (*Cd68*, *Cx3cr1*), and CSF1R‐signaling ligands (*Csf1*, *Il34*) (Figure 1N). Gene Ontology enrichment highlighted complement receptor activity, complement activation, and synaptic pruning related biological processes (Figure 1O), while KEGG pathway analysis revealed enrichment for complement cascades, cytokine‐cytokine receptor interactions, and MAPK signaling (Figure 1P). Collectively, these results show that HI triggers mast‐cell activation and tryptase elevation associated with early neurological deficits, accompanied by broad transcriptomic reprogramming encompassing complement driven innate immunity and MAPK mediated neuroinflammation.

**FIGURE 1:**
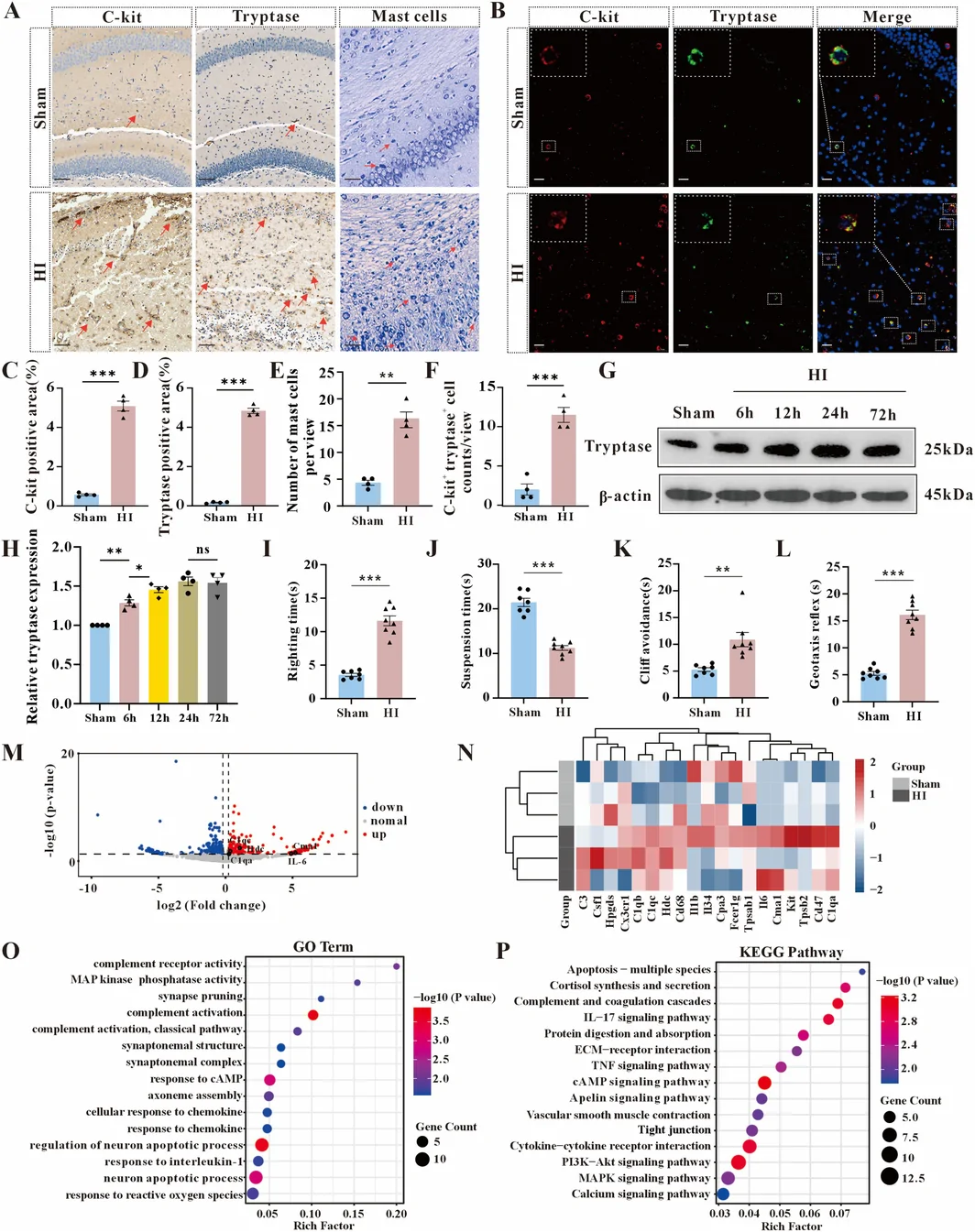
HI insult increases hippocampal mast cell‐associated c‐Kit/tryptase signals and tryptase expression, which are associated with early neurobehavioral impairment. (A) Representative c‐Kit and tryptase immunohistochemical staining and H&E‐stained hippocampal sections from Sham and HI rats. Red arrows indicate representative mast cell associated signals or morphologically identified mast cells. Scale bars = 50 μm. (B) Representative immunofluorescence images showing c‐Kit (red), tryptase (green), DAPI (blue), and merged images, with enlarged views of selected regions. Scale bars = 100 μm. (C–F) Quantification of c‐Kit positive area, tryptase positive area, morphologically identified mast cells, and c‐Kit^+^/tryptase^+^ cells, respectively. (G, H) Representative immunoblot and quantification of tryptase in hippocampal tissue collected at 6, 12, 24, and 72 h after HI. (I–L) Early neurobehavioral assessments performed 24 h after HI injury, including righting‐reflex latency (I), forelimb‐suspension time (J), cliff‐avoidance time (K), and negative‐geotaxis latency (L). (M) Volcano plot of differentially expressed genes in bulk hippocampal tissue from HI and Sham rats. Red dots indicate upregulated genes, blue dots indicate downregulated genes, and gray dots indicate genes without significant differential expression. (N) Heatmap of selected genes associated with complement signaling, mast cell associated proteases and receptors, inflammatory signaling, and myeloid/microglia associated pathways. Group annotations are shown separately from the gene‐expression color scale. (O, P) Gene Ontology (GO) enrichment analysis and Kyoto Encyclopedia of Genes and Genomes (KEGG) pathway enrichment analysis of differentially expressed genes in bulk hippocampal tissue. Data are presented as mean ± SEM, *p < 0.05, **p < 0.01, ***p < 0.001. *n* = 4 animals per group for histological and biochemical analyses and *n* = 8 animals per group for acute behavioral tests.

### 3.2 HI Insult Promotes Abnormal Microglia Associated Synaptic Pruning

To determine whether HI injury is associated with aberrant synaptic pruning involving complement and chemokine signaling, we examined key molecular and cellular features of this process. Complement proteins C1q and C3 serve as “eat‐me” signals that label synapses for elimination by microglia [27]. CX3CR1 is a fractalkine receptor involved in microglia–neuron communication, adhesion, and chemotaxis through neuronal CX3CL1, whereas CD68 is a lysosomal marker commonly used to identify microglia with a phagocytosis‐associated phenotype [27, 28]. We found that compared with sham controls, HI injured rats displayed markedly higher numbers of C1q^+^/NeuN^+^ and C3^+^/NeuN^+^ cells (Figure 2A,B,E,H), accompanied by significantly elevated relative fluorescence intensities of C1q and C3 (Figure 2C,D,F,G). For microglial phagocytic markers, HI rats showed increased counts of Iba1^+^/CD68^+^ and Iba1^+^/CX3CR1^+^ microglia (Figure 2I,J,M,P), along with enhanced relative fluorescence intensities of CD68 and CX3CR1 (Figure 2K,L,N,O). Elevated Iba1 colocalized CX3CR1 signal reflected upregulated microglial CX3CR1 expression/localization, implying perturbed CX3CL1‐CX3CR1 signaling. In parallel, increased CD68 expression denoted a lysosome‐enriched phagocytic phenotype. Collectively, these data demonstrate augmented complement deposition and microglial phenotypic shifts linked to synaptic pruning after HI.

**FIGURE 2:**
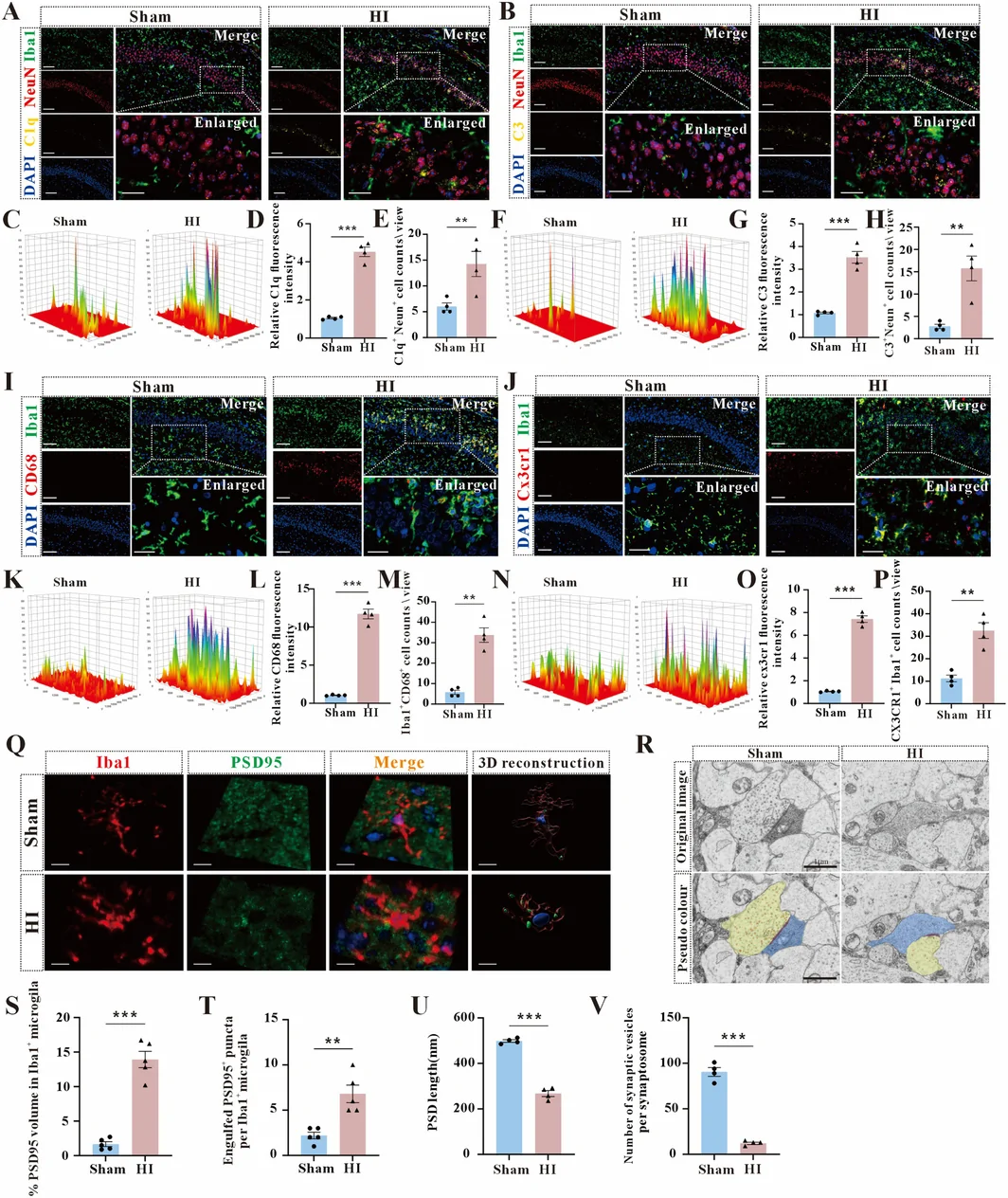
HI insult increases synaptic complement labeling, microglia associated CD68 and CX3CR1 signals, and engulfment of synaptic material. (A) Representative immunofluorescence images showing C1q (yellow), NeuN (red), Iba1 (green), and DAPI (blue) in the hippocampus of Sham and HI rats, together with enlarged views and single‐channel images. (B) Representative immunofluorescence images showing C3 (yellow), NeuN (red), Iba1 (green), and DAPI (blue), together with enlarged views and single‐channel images. (C–E) Fluorescence‐intensity surface plots of C1q, quantification of C1q fluorescence intensity, and quantification of C1q^+^/NeuN^+^ cells, respectively. (F–H) Corresponding analyses for C3. (I) Representative immunofluorescence images showing Iba1 (green), CD68 (red), and DAPI (blue), together with enlarged views and single‐channel images. (J) Representative immunofluorescence images showing Iba1 (green), CX3CR1 (red), and DAPI (blue), together with enlarged views and single‐channel images. (K–M) Fluorescence‐intensity surface plots of CD68, quantification of CD68 fluorescence intensity, and quantification of CD68^+^/Iba1^+^ cells, respectively. (N–P) Corresponding analyses for CX3CR1. Scale bar = 100 μm in panel A, B, I, J. (Q) Representative confocal images of Iba1^+^ microglia (red), PSD95 (green), and DAPI (blue) in the hippocampus, together with 3D reconstructions. Scale bars = 10 μm. (R) Representative transmission electron micrographs and pseudo‐colored images of hippocampal synaptic structures. Scale bar = 1 μm (S, T). Quantification of PSD95 volume and PSD95 puncta internalized within Iba1^+^ microglia. (U, V) Quantification of postsynaptic density length and synaptic vesicle number per synaptosome. Data are presented as mean ± SEM, *p < 0.05, **p < 0.01, ***p < 0.001. *n* = 4 animals per group.

To obtain direct evidence for microglia‐dependent synaptic engulfment, we performed 3D reconstruction of immunofluorescence labeled Iba1^+^ microglia and the postsynaptic marker PSD95 using Imaris software. Quantitative 3D analysis revealed a significantly increased volume of PSD95 signal internalized within Iba1^+^ microglia in HI rats compared with sham rats, providing direct morphological evidence of enhanced engulfment of postsynaptic components (Figure 2Q,S,T). Furthermore, ultrastructural analysis showed that HI injury reduced the number of presynaptic vesicles and shortened the postsynaptic density compared with the sham group (Figure 2R,U,V). Taken together, these results suggest that HI provokes pathological microglia‐mediated synaptic pruning, manifested by robust complement deposition, a CD68 positive phagocytic microglial state, increased postsynaptic engulfment, and disrupted synaptic ultrastructure.

### 3.3 PAR‐2/MAPK/NF‐κB Signaling Contributes to Microglia Mediated Synaptic Pruning

Accumulating evidence indicates that PAR‐2 acts as a critical signaling bridge linking mast cell activation to microglial inflammatory responses. Given the robust upregulation of mast cell‐derived tryptase following HI injury in our model, we next examined the cellular distribution and expression changes of PAR‐2 in hippocampal neurons, astrocytes, and microglia. Single‐channel fluorescence quantification demonstrated a significant elevation in overall PAR‐2 immunoreactivity in the HI group relative to sham controls (Figure 3A,B). Notably, the colocalization levels of PAR‐2 with NeuN^+^ neurons and GFAP^+^ astrocytes remained unaltered after HI injury (Figure 3A,C,D). In contrast, both PAR‐2 fluorescence intensity and the abundance of PAR‐2^+^/Iba1^+^ double‐positive microglia were markedly increased in HI rats (Figure 3E–H). These results demonstrate that HI specifically induces PAR‐2 upregulation predominantly in microglia, rather than in neurons or astrocytes.

**FIGURE 3:**
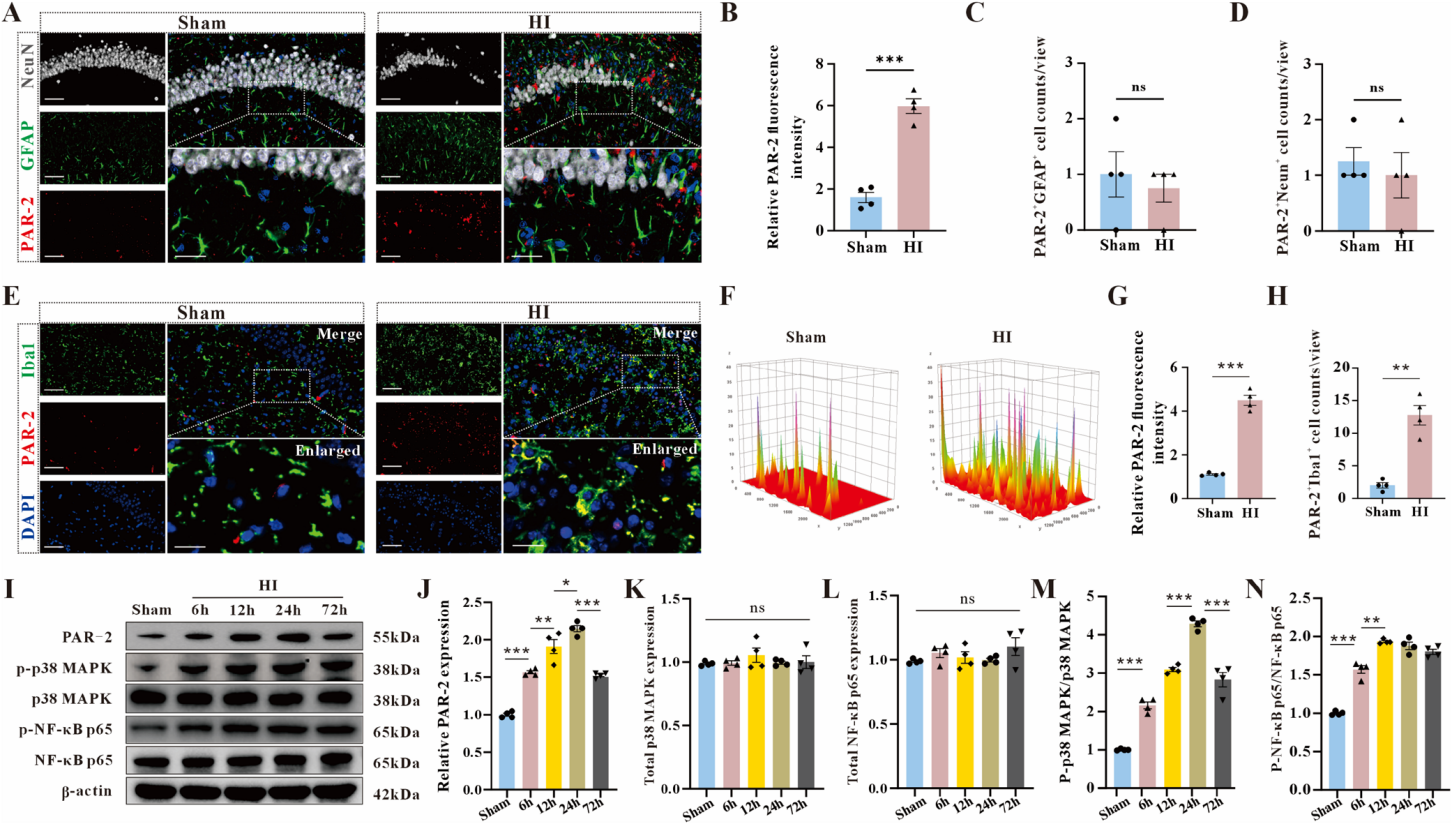
HI insult increases PAR‐2 associated signals in microglia and activates the MAPK/NF‐κB pathway. (A) Representative immunofluorescence images showing NeuN (white), GFAP (green), PAR‐2 (red), DAPI (blue), and merged images in sham and HI groups. Scale bar = 100 μm. (B–D) Quantification of PAR‐2 fluorescence intensity, PAR‐2^+^ /GFAP^+^ cells, and PAR‐2^+^/NeuN^+^ cells, respectively. (E) Representative immunofluorescence images showing Iba1 (green), PAR‐2 (red), DAPI (blue), and merged images in sham and HI groups. Scale bar = 100 μm. (F, G) Fluorescence‐intensity surface plots of the PAR‐2 channel and quantification of PAR‐2 fluorescence intensity. (H) Quantification of PAR‐2^+^/Iba1^+^ cells per view in sham versus HI groups. (I) Representative immunoblots of PAR‐2, p‐p38 MAPK, p38 MAPK, p‐NF‐κB p65, NF‐κB p65 and β‐actin in hippocampal tissue. (J–L) Quantification of PAR‐2, p38 MAPK and NF‐κB p65 normalized to β‐actin. (M, N) Quantification of p‐p38 MAPK/p38 MAPK and p‐NF‐κB p65/NF‐κB p65 ratios, respectively. *n* = 4 animals per group. Data are presented as mean ± SEM, *p < 0.05, **p < 0.01, ***p < 0.001.

We further characterized the activation status of the downstream MAPK/NF‐κB signaling cascade. Western blot analysis revealed significantly elevated PAR‐2 protein expression, as well as increased phosphorylation ratios of p38 MAPK and NF‐κB p65 after HI injury. These signaling alterations peaked at 24 h post‐insult, while the total protein levels of p38 MAPK and NF‐κB p65 remained stable across groups (Figure 3I–N). The temporal activation pattern of PAR‐2 and its downstream signaling cascade was consistent with the dynamic elevation of mast cell tryptase, implying a potential regulatory axis of mast cell tryptase/PAR‐2/MAPK/NF‐κB in HI brain injury.

To further validate the functional role of PAR‐2 signaling in mediating HI triggered microglial activation and pathological synaptic pruning, we pharmacologically inhibited PAR‐2 activity via pretreatment with the PAR‐2 antagonist F‐NH2 prior to HI modeling. Compared with the vehicle treated HI group, F‐NH2 treatment significantly reduced PAR‐2 fluorescence intensity and decreased the number of PAR‐2^+^/Iba1^+^ microglia (Figure S3A–D). In addition, F‐NH2 treatment markedly reversed the HI induced hyperphosphorylation of p38 MAPK and NF/κB p65, with no obvious influence on their total protein expression (Figure S3E–J). Moreover, PAR‐2 inhibition mitigated microglial phagocytic activation, as evidenced by reduced CD68 expression, and partially restored the levels of synaptic structural proteins PSD95 and SYP (Figure S3K–N). Consistent with these molecular changes, 3D reconstruction analysis further confirmed that F‐NH2 treatment reduced both the quantity and volume of PSD95 puncta engulfed by Iba1^+^ microglia (Figure S5O–Q). Collectively, these pharmacological results solidify that PAR‐2/MAPK/NF‐κB signaling is a crucial upstream pathway driving microglial activation and excessive synaptic engulfment following neonatal HI injury.

### 3.4 Microglia‐Targeted F2rl1 Silencing Attenuates p38 MAPK/NF‐κB Signaling and Synaptic Engulfment

To further clarify the cell specific function of microglial PAR‐2 (encoded by *F2rl1*) in mediating HI induced inflammatory signaling and pathological synaptic engulfment, we specifically knocked down microglial *F2rl1* via stereotactic injection of AAV9‐CX3CR1‐shF2rl1, with AAV9‐CX3CR1‐shNC serving as the negative control (Figure 4A,B). Successful *F2rl1* knockdown was verified by the markedly reduced *F2rl1* mRNA level in the HI+shF2rl1 group relative to the HI + shNC group (Figure 4C). Consistent with the transcriptional suppression of *F2rl1*, microglial *F2rl1* silencing significantly downregulated PAR‐2 protein expression and mitigated HI induced hyperphosphorylation of p38 MAPK and NF‐κB p65, while the total protein levels of p38 MAPK and NF‐κB p65 remained unchanged. In addition, *F2rl1* knockdown efficiently reduced the expression of the phagocytic marker CD68 (Figure 4D–G). Moreover, the loss of synaptic proteins PSD95 and SYP following HI injury was partially rescued by microglial *F2rl1* inhibition (Figure 4D,E). Consistently, 3D reconstruction analysis demonstrated a prominent reduction in the volume of PSD95 puncta internalized within Iba1^+^ microglia after *F2rl1* silencing (Figure 4H,I). Taken together, these cell targeted knockdown results confirm that microglial PAR‐2 is a potential regulator of HI triggered p38 MAPK/NF‐κB signaling activation, CD68 dependent phagocytic phenotypic transformation, and excessive microglia mediated synaptic engulfment.

**FIGURE 4:**
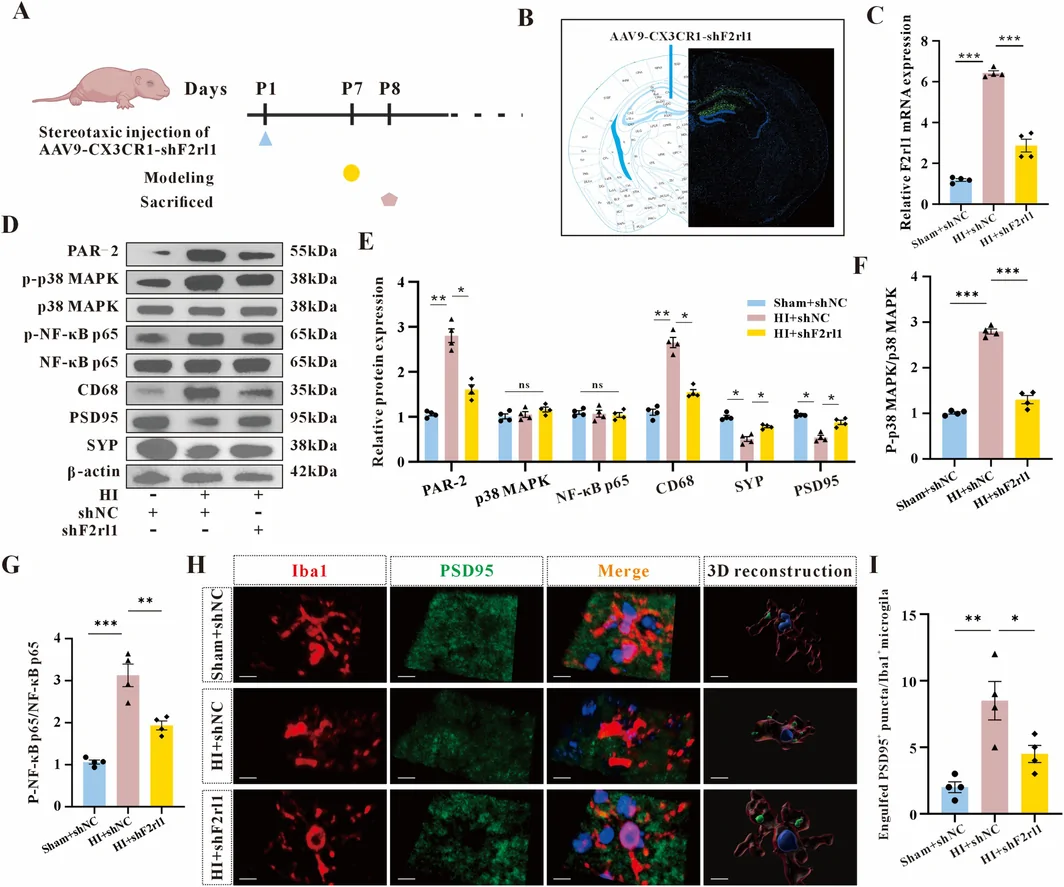
Microglia‐targeted F2rl1 silencing attenuates HI‐associated PAR‐2 upregulation, downstream MAPK/NF‐κB signaling activation, and excessive microglial synaptic engulfment. (A) Schematic illustration of the experimental design. Neonatal rats received stereotaxic hippocampal injection of AAV9‐CX3CR1‐shNC or AAV9‐CX3CR1‐shF2rl1 on postnatal Day 1 (P1). HI injury or sham surgery was performed on P7, and hippocampal tissues were collected on P8 for subsequent analyses. (B) Schematic representation of the injection site and a representative coronal brain section showing the AAV associated reporter signal in the hippocampus. (C) Relative F2rl1 mRNA expression in hippocampus determined by quantitative real‐time PCR. (D) Representative immunoblots of PAR‐2, p‐p38 MAPK, p38 MAPK, p‐NF‐κB p65, NF‐κB p65, CD68, PSD95, SYP, and β‐actin in hippocampal tissue. (E) Quantification of PAR‐2, p38 MAPK, NF‐κB p65, CD68, PSD95, and SYP normalized to β‐actin. (F, G) Quantification of p‐p38 MAPK/p38 MAPK and p‐NF‐κB p65/NF‐κB p65 ratios, respectively. (H, I) Representative confocal images of Iba1^+^ microglia (red), PSD95 (green), and DAPI (blue) in the hippocampus, together with 3D reconstructions. Scale bars = 10 μm. Quantification of PSD95 puncta internalized within Iba1^+^ microglia was performed to evaluate microglia‐mediated synaptic engulfment. *n* = 4 animals per group. Data are presented as mean ± SEM, *p < 0.05, **p < 0.01, ***p < 0.001.

### 3.5 Tryptase Related Signaling Contributes to PAR‐2/MAPK/NF‐κB Activation and Microglial Synaptic Engulfment

Mast cell‐derived tryptase has been reported to interact with microglial PAR‐2 and trigger neuroinflammatory responses. To further determine whether tryptase acts as an upstream regulator of the PAR‐2/MAPK/NF‐κB cascade and mediates HI‐induced pathological synaptic pruning, we pharmacologically inhibited endogenous tryptase activity using the selective tryptase inhibitor APC366. Immunofluorescence analysis revealed that APC366 treatment effectively abolished the HI‐induced upregulation of PAR‐2 immunoreactivity and reduced the abundance of PAR‐2^+^/Iba1^+^ microglia (Figure 5A–D). Consistent with these phenotypic changes, Western blot results demonstrated that APC366 markedly decreased the protein levels of tryptase and PAR‐2 and suppressed the hyperphosphorylation of p38 MAPK and NF‐κB p65 following HI injury (Figure 5E–H). Moreover, tryptase inhibition reduced CD68 expression, restored the downregulated synaptic proteins PSD95 and SYP, and diminished the volume and abundance of PSD95 puncta engulfed by Iba1^+^ microglia (Figure 5I–M). Notably, neither APC366 nor F‐NH2 intervention altered the basal levels of PAR‐2, MAPK/NF‐κB signaling, CD68, PSD95, or SYP under sham conditions, excluding non‐specific effects of drug treatment (Figure S4).

**FIGURE 5:**
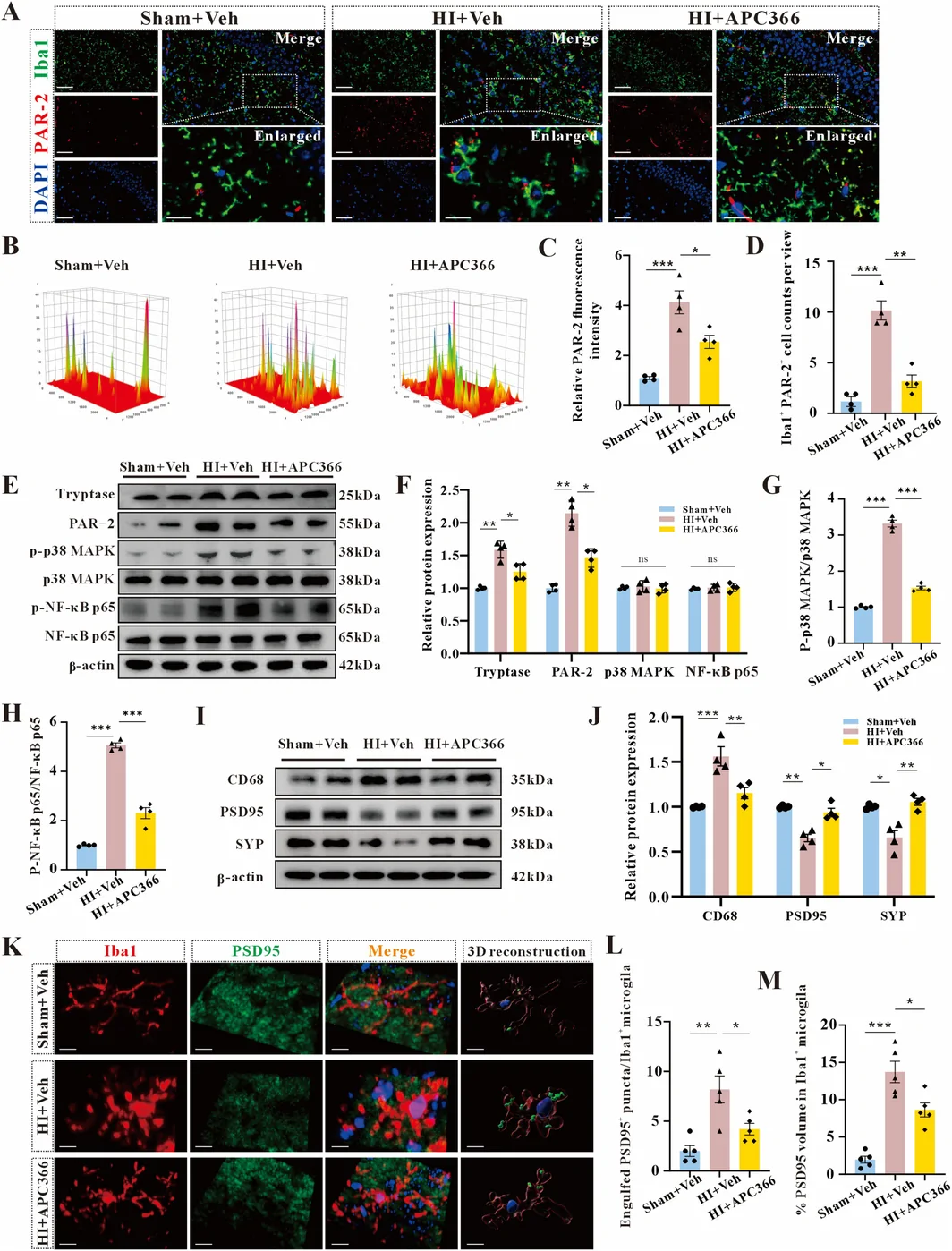
Tryptase inhibition with APC366 is associated with reduced microglial PAR‐2 signal, attenuated MAPK/NF‐κB activation, and reduced synaptic engulfment after HI. (A) Representative immunofluorescence images showing Iba1 (green), PAR‐2 (red), and DAPI (blue) of Sham+Veh, HI+Veh, and HI+APC366 rats, together with enlarged views and single‐channel images. Scale bar = 100 μm. (B, C) Fluorescence‐intensity surface plots of PAR‐2 and quantification of PAR‐2 fluorescence intensity, respectively. (D) Quantification of Iba1^+^/PAR‐2^+^ cells per field of view. (E) Representative immunoblots of tryptase, PAR‐2, p‐p38 MAPK, p38 MAPK, p‐NF‐κB p65, NF‐κB p65, and β‐actin in hippocampal tissue from Sham+Veh, HI+Veh, and HI+APC366 rats. (F) Quantification of tryptase, PAR‐2, p38 MAPK, and NF‐κB p65 normalized to β‐actin. (G, H) Quantification of p‐p38 MAPK/p38 MAPK and p‐NF‐κB p65/NF‐κB p65 ratios, respectively. (I) Representative immunoblots of CD68, PSD95, SYP, and β‐actin. (J) Quantification of CD68, PSD95, and SYP normalized to β‐actin. (K) Representative confocal images of Iba1^+^ microglia (red) and PSD95 (green), together with merged images and 3D reconstructions. Scale bars = 10 μm. (L, M) Quantification of PSD95 puncta engulfed by Iba1^+^ microglia and PSD95 volume within Iba1^+^ microglia, respectively. Data are presented as mean ± SEM. *n* = 4 animals per group.

In vitro OGD/R experiments using BV2 microglia further validated the regulatory axis between tryptase and PAR‐2 signaling. Tryptase stimulation markedly increased the expression of PAR‐2, CD68, and elevated the phosphorylation levels of p38 MAPK and NF‐κB p65 under OGD/R conditions, whereas pharmacological blockade of PAR‐2 with F‐NH2 efficiently reversed these tryptase‐mediated pro‐inflammatory and phagocytic alterations (Figure S5A–F). In contrast, the total protein levels of p38 MAPK and NF‐κB p65 showed no significant differences among these groups (Figure S5G,H). Collectively, these in vivo and in vitro findings demonstrate that mast cell‐derived tryptase serves as a critical upstream trigger for microglial PAR‐2 activation, which initiates downstream MAPK/NF‐κB signaling and ultimately drives excessive microglial synaptic engulfment after HI injury.

### 3.6 APC366 Attenuates Synaptic Damage and Improves Long‐Term Cognitive Function After HI Insult

Given the beneficial effects of tryptase inhibition on suppressing microglial overactivation and synaptic loss, we further explored whether APC366 could protect synaptic ultrastructure and rescue long‐term cognitive deficits following HI injury. Golgi staining analysis showed that HI injury markedly reduced dendritic complexity and spine density, while APC366 treatment significantly increased dendritic intersection numbers and spine density, restoring these morphological parameters to levels comparable to those in sham animals (Figure 6A–C). Consistent with the dendritic remodeling results, transmission electron microscopy (TEM) further confirmed synaptic structural protection by APC366. Compared with the vehicle‐treated HI group, APC366 intervention increased the number of presynaptic vesicles and elongated the postsynaptic density length, effectively ameliorating HI‐induced synaptic damage (Figure 6D–F).

**FIGURE 6:**
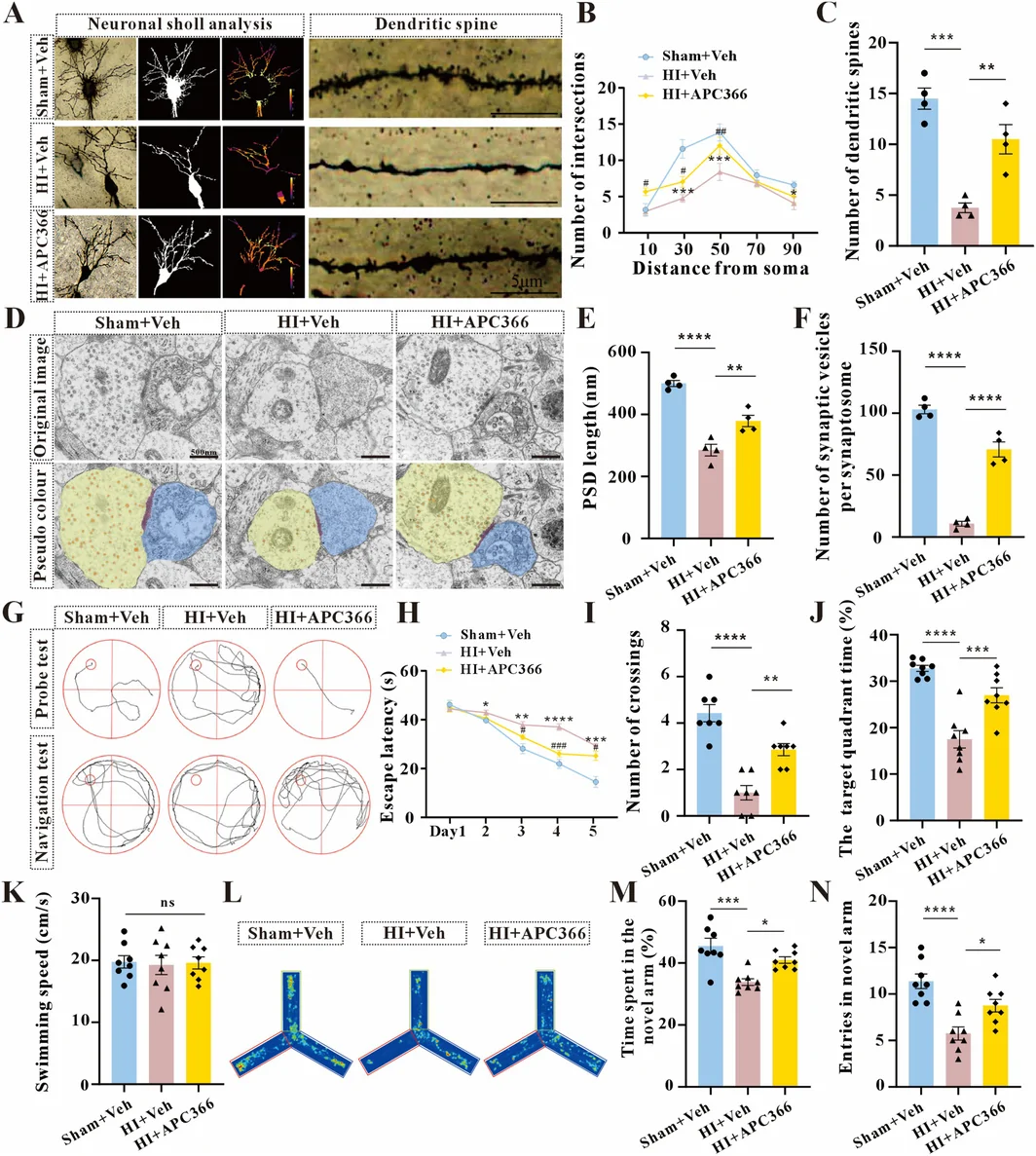
APC366 restores dendritic spine density, synaptic ultrastructure, and spatial memory performance in HI rats. (A) Representative images of neuronal morphology (left: Neuronal shell analysis, right: Dendritic spine imaging) from Sham+Veh, HI+Veh, and HI+APC366 groups. Scale bars = 5 μm. (B) Sholl analysis showing the number of dendritic intersections as a function of distance from the soma. (C) Quantification of dendritic spine density per 10 μm dendrite in each group. (D) Representative transmission electron micrographs (upper) and pseudo color illustrations (lower) displaying postsynaptic densities (PSD) and synaptic vesicles in hippocampal CA1 regions. Scale bars = 500 nm. (E) Quantification of PSD length in hippocampal synapses. (F) Quantification of synaptic vesicle number per presynaptic terminal. (G) Probe test traces and navigation test swim paths in the Morris water maze for each experimental group. (H) Escape latency curves during training days in the Morris water maze task. (I) Quantification of platform area crossings during the probe test. (J) Percentage of time spent in the target quadrant during the probe test. (K) Swimming speed measurement among groups in the Morris water maze. (L) Representative heatmaps of swim patterns in the Y‐maze novel arm test. (M) Quantification of the percentage of time spent in the novel arm during the Y‐maze test. (N) Quantification of the number of entries into the novel arm in the Y‐maze. Data are presented as mean ± SEM. *n* = 4 animals per group for histological and biochemical analyses and *n* = 8 animals per group for behavioral tests.

We subsequently evaluated long‐term cognitive function via the MWM and Y‐maze tests. During the MWM training phase, HI rats exhibited significantly prolonged escape latencies, indicating impaired spatial learning ability, whereas APC366 treatment substantially shortened escape latency and improved learning performance (Figure 6H). In the probe trial, APC366 treated HI rats displayed increased target platform crossings and prolonged residence time in the target quadrant, reflecting improved spatial memory retention (Figure 6I,J). No significant differences in swimming speed were observed across groups, ruling out the interference of motor function (Figure 6K). The Y‐maze test further verified the cognitive protective effects of APC366. HI induced deficits in novel arm exploration, including reduced novel arm entries and shorter exploration time, were significantly rescued by APC366 administration (Figure 6L–N). Taken together, these results demonstrate that tryptase inhibition by APC366 preserves hippocampal synaptic integrity and exerts long‐term protective effects on cognition after neonatal HI injury.

## 4 Discussion

This study identifies MC‐derived tryptase as a potential upstream trigger of excessive synaptic pruning after neonatal HI. HI insult increased hippocampal MC accumulation and tryptase production and further activated PAR‐2/p38 MAPK/NF‐κB signaling in microglia. This signaling activation was associated with C1q/C3‐dependent synaptic tagging and increased microglial engulfment of PSD95‐containing postsynaptic fragments. Microglia‐targeted F2rl1 silencing reduced pathway activation and synaptic engulfment, confirming the functional requirement of microglial PAR‐2 in this pathological cascade. Pharmacological blockade of either tryptase or PAR‐2 yielded comparable neuroprotective phenotypes. Moreover, tryptase inhibition attenuated synaptic damage and rescued long‐term cognitive impairments. These findings expand the classical view of mast cells as purely peripheral immune cells, demonstrating their capacity to modulate microglia‐dependent synaptic homeostasis within the injured neonatal brain.

MC are increasingly recognized as resident innate immune cells that rapidly respond to injury and modulate neuroinflammatory processes, including changes in blood brain barrier integrity observed in various CNS disorders [13, 29]. Consistent with this role, hippocampal tryptase increased within 6 h after HI and remained elevated for up to 24 h, indicating that the tryptase response begins early and persists during the acute phase of injury. Increased c‐Kit and tryptase signals, together with a greater number of morphologically identified hippocampal MC, further linked this response to MC changes after HI. MC counts and tryptase levels were also associated with acute neurological deficits, suggesting that these early immune changes are related to the severity of neurological dysfunction. Together, the early time course, histological findings, and associations with neurological deficits support a role for MC‐derived tryptase in the early pathological response to neonatal HI.

MC play a pivotal role as “pioneers” of the immune response during the initial stages of brain injury or inflammation by rapidly releasing mediators, such as tryptase and histamine, which activate microglia both directly and indirectly [30, 31]. Under physiological conditions, microglia eliminate redundant or dysfunctional synaptic connections through precise pruning, which is essential for synaptic development and plasticity [16, 17, 19]. However, in the pathological context of HI injury, we observed four features indicative of aberrant rather than developmental pruning. First, complement labeling was markedly increased, with neuron associated C1q and C3 signals far exceeding those observed during normal developmental pruning and indicating excessive complement associated synaptic tagging. Second, RNA analysis and immunostaining indicated an enhanced microglial phagocytic state and altered microglia–neuron communication after HI. Increased CD68 in Iba1^+^ microglia supported greater lysosomal and phagocytic activity. The rise in CX3CR1 signal reflected changes in CX3CL1–CX3CR1 mediated neuron–microglia communication during synaptic remodeling [27, 32]. Third, this phagocytic state was accompanied by a substantial increase in PSD95 positive material internalized within microglia, reflecting non‐selective engulfment rather than refinement of weak synapses. Finally, ultrastructural analysis revealed synaptic damage, including reduced presynaptic vesicles and shortened postsynaptic densities, consistent with synaptic loss rather than physiological remodeling. Collectively, these findings distinguish pathological pruning from homeostatic developmental remodeling and provide a mechanistic basis for the observed cognitive deficits following neonatal HI insult.

PAR‐2 is a pivotal mediator of MC–microglia interactions. Unlike PAR‐1, PAR‐3, and PAR‐4, which are primarily activated by thrombin, PAR‐2 can be activated by mast cell tryptase [22, 30, 31]. In line with this, we observed PAR‐2 fluorescence and PAR‐2^+^/Iba1^+^ microglia increased markedly after HI. This pattern was reversed according to pharmacological blockade of PAR‐2 by F‐NH2. Compared with sham rats, F‐NH2 treatment attenuated microglial PAR‐2 upregulation and suppressed activation of the MAPK/NF‐κB pathway after HI. These data support a required role for PAR‐2 in HI‐induced activation of the MAPK/NF‐κB pathway. Consistent with this, F‐NH2 reduced CD68 expression, restored PSD95 and SYP levels, and microglial engulfment of PSD95. Microglia‐targeted F2rl1 silencing reproduced these effects, identifying microglial PAR‐2 as a key link between tryptase signaling and pathological synaptic engulfment. While microglia‐targeted F2rl1 silencing supports a key role for microglial PAR‐2 in HI‐associated synaptic engulfment, the effects of systemic pharmacological PAR‐2 inhibition may also involve PAR‐2 expressed by other CNS cell types. Unlike previous studies that have mainly linked PAR‐2 to neuroinflammation in neurodegenerative disorders [33, 34, 35], our findings extend its role to aberrant synaptic pruning in neonatal HI, thereby broadening its relevance in central nervous system injury.

MC‐derived tryptase, an endogenous activator of PAR‐2, plays an important role in neuroinflammation. Previous studies have shown that tryptase activates PAR‐2 in microglia and astrocytes, promoting the release of pro‐inflammatory cytokines such as IL‐6 and TNF‐α [15, 36]. In a cardiac arrest‐induced global ischemia model, increased tryptase and PAR‐2 expression was associated with neurological deficits and hippocampal neuronal degeneration, whereas APC366 treatment exerted neuroprotective effects [8]. Consistent with these findings, APC366 reduced PAR‐2/p38 MAPK/NF‐κB signaling and microglial engulfment of PSD95 positive material after neonatal HI. Complementary BV‐2 experiments further showed that exogenous tryptase activated PAR‐2/p38 MAPK/NF‐κB signaling and increased CD68 expression under OGD/R conditions, whereas F‐NH2 attenuated these responses. These findings support a direct role for tryptase in activating PAR‐2‐dependent signaling in microglia. APC366 also preserved dendritic complexity, spine density, and synaptic ultrastructure. These structural benefits were accompanied by improved long‐term spatial learning and memory after HI. Together, these findings identify MC‐derived tryptase as an upstream driver of PAR‐2‐dependent synaptic injury and suggest that early inhibition of MC–microglia signaling may reduce long‐term neurological impairment after neonatal HI.

This study has several limitations. We did not characterize the specific microglial subpopulation mediating this pathological cascade, nor did we explore whether this signaling axis operates comparably in the mature brain. In addition, although microglia‐targeted *F2rl1* silencing strengthens causal inference, systemic pharmacological PAR‐2 inhibition cannot rule out contributions from other CNS cell types. Moreover, the study was not statistically powered to evaluate potential sex‐dependent effects.

In conclusion, our present findings demonstrate that MC‐derived tryptase drives aberrant microglia‐mediated synaptic pruning in neonatal HIE. Microglial *F2rl1* knockdown, as well as pharmacological inhibition of PAR‐2 or tryptase, reduced p38 MAPK/NF‐κB activation and excessive synaptic engulfment. Tryptase inhibition additionally maintained synaptic integrity and rescued cognitive performance. Early therapeutic targeting of MC‐microglia crosstalk may represent a promising strategy to limit long‐term neurological deficits following neonatal HI.

## Author Contributions

X.C. and X.Z. conceived and designed the experiments. Y.Y., Y.Z., J.Y., Y.H., Y.L., C.T., J.W., and Y.L. conducted the experiments. Y.Y., D.C., and J.Y. analyzed the data. A.C., D.D., and F.X. revised the manuscript for language and content. X.C., X.Z., A.C., Y.Y., D.C., and J.Y. wrote and edited the paper.

## Funding

This study was supported by funding from the National Natural Science Foundation of China (Nos. 82571351 and 82471278), Fujian Research and Training Grants for Young and Middle‐aged Leaders in Healthcare, the Natural Science Foundation of Fujian Province (No. 2024J010035), and the Joint Funds for the Innovation of Science and Technology of Fujian Province (Nos. 2023Y9315, 2023Y9282 and 2025Y9056), and the Medical Innovations Topic of Fujian Province, China (No. 2024CXA002).

## Ethics Statement

All animal experiments were approved by the Animal Care and Use Committee at Fujian Provincial Hospital (Fuzhou, China) (Approval No: IACUC‐FPH‐PZ‐20250304).

## Consent

All the co‐authors and participants have given their consent for publication.

## Conflicts of Interest

The authors declare no conflicts of interest.

## Supporting information


**Figure S1:** Establishment and characterization of the neonatal HI rat model. (A, B) Representative TTC‐stained coronal sections showing infarcted (white) regions in the ipsilateral hemisphere of HI rats compared with sham controls. Quantification reveals that infarction volume accounts for approximately 30% of the hippocampal region. (C, D) Nissl staining of hippocampal CA1 regions indicates a marked reduction in surviving neurons following HI compared with shams. (E, F) Representative TUNEL staining images demonstrate increased apoptotic cell death in hippocampal tissues from HI rats relative to sham controls. Quantification of TUNEL‐positive cells confirms significant elevation of apoptosis post‐HI. *n* = 4 animals per group. Statistical significance was assessed using two‐tailed Student's *t*‐test or one‐way ANOVA with appropriate post hoc tests. Significance levels are shown in the graphs. Scale bars = 50 μm.
**Figure S2:** Increased mast cells and tryptase correlate with acute neurological impairment in HI mice. (A–H) Correlation analysis between MCs counts (*x*‐axis) and individual behavioral scores (*y*‐axis): (A) righting time, (B) cliff‐avoidance time, (C) geotaxis latency, (D) suspension time. (E–H) Simple linear regressions between relative tryptase expression (*x*‐axis) and the same behavioral parameters: (E) righting time, (F) cliff‐avoidance time, (G) geotaxis latency, (H) suspension time. Shaded areas indicate 95% confidence intervals; regression equations and coefficients of determination (*R* ^2^) are displayed on each graph.
**Figure S3:** F‐NH2 attenuates HI‐associated PAR‐2 signaling and microglial synaptic engulfment. (A) Representative immunofluorescence images showing Iba1 (green), PAR‐2 (red), and DAPI (blue) in the hippocampus, together with enlarged views and single‐channel images. Scale bars = 100 μm (B–D) Quantification of Iba1^+^/PAR‐2^+^ cells, corresponding fluorescence‐intensity surface plots of PAR‐2, and PAR‐2 fluorescence intensity, respectively. (E) Representative immunoblots of PAR‐2, p‐p38 MAPK, p38 MAPK, p‐NF‐κB p65, NF‐κB p65, and β‐actin in hippocampal tissue. (F, I, J) Quantification of PAR‐2, p38 MAPK, and NF‐κB p65 normalized to β‐actin. (G, H) Quantification of p‐p38 MAPK/p38 MAPK and p‐NF‐κB p65/NF‐κB p65 ratios, respectively. (K) Representative immunoblots of CD68, PSD95, SYP and β‐actin. (L–N) Quantification of CD68, PSD95 and SYP expression normalized to β‐actin, respectively. (O) Representative confocal images of Iba1^+^ microglia (red) and PSD95 (green), together with merged images and 3D reconstructions. Scale bars = 10 μm (P, Q) Quantification of PSD95 puncta engulfed by Iba1^+^ microglia and PSD95 volume within Iba1‐positive microglia, respectively. *n* = 4 animals per group. Data are presented as mean ± SEM, *p < 0.05, **p < 0.01, ***p < 0.001. Statistical significance was assessed using one‐way ANOVA with appropriate post hoc tests. Significance levels are shown in the graphs.
**Figure S4:** Sham treatment with F‐NH2 or APC366 does not significantly alter PAR‐2/MAPK/NF‐κB signaling or synaptic and microglia‐associated protein expression. (A) Representative immunoblots of PAR‐2, p‐p38 MAPK, p38 MAPK, p‐NF‐κB p65, NF‐κB p65, CD68, PSD95, SYP and β‐actin in hippocampal tissue. (B) Quantification of the p‐p38 MAPK/p38 MAPK ratio. (C) Quantification of the p‐NF‐κB p65/NF‐κB p65 ratio. (D) Quantification of PAR‐2, p38 MAPK, NF‐κB p65, CD68, PSD95 and SYP normalized to β‐actin. *n* = 4 animals per group. Data are presented as mean ± SEM, *p < 0.05, **p < 0.01, ***p < 0.001. Statistical significance was assessed using one‐way ANOVA with appropriate post hoc tests. Significance levels are shown in the graphs.
**Figure S5:** Exogenous tryptase potentiates OGD/R‐associated CD68 expression and p38 MAPK/NF‐κB phosphorylation in BV‐2 cells, which are attenuated by F‐NH2. (A) Representative immunofluorescence images of BV‐2 cells stained for Iba1 (green), CD68 (red), and DAPI (blue), together with merged images and fluorescence‐intensity surface plots under the indicated conditions: Ctrl+Veh, OGD/*R* + Veh, OGD/*R* + F‐NH2, OGD/*R* + tryptase, and OGD/*R* + tryptase+F‐NH2. Scale bars = 10 μm. (B) Representative immunoblots of CD68, p‐p38 MAPK, p‐NF‐κB p65 in BV‐2 cells exposed to the indicated conditions. (C) Quantification of PAR‐2 expression normalized to β‐actin. (D) Quantification of the p‐p38 MAPK/p38 MAPK ratio. (E) Quantification of the p‐NF‐κB p65/NF‐κB p65 ratio. (F) Quantification of CD68 expression normalized to β‐actin. (G) Quantification of total p38 MAPK expression normalized to β‐actin. (H) Quantification of total NF‐κB p65 expression normalized to β‐actin. *n* = 4 per group. Data are presented as mean ± SEM, *p < 0.05, **p < 0.01, ***p < 0.001. Statistical significance was assessed using one‐way ANOVA with appropriate post hoc tests. Significance levels are shown in the graphs.

**Table S1:** Antibodies used in this study.

## Data Availability

Data supporting the findings of this study are available upon reasonable request to the corresponding author.

## References

[1] E. Chakkarapani , L. S. de Vries , D. M. Ferriero , and A. J. Gunn , “Neonatal Encephalopathy and Hypoxic‐Ischemic Encephalopathy: The State of the Art,” Pediatric Research 98, no. 7 (2025): 2444–2458. 10.1038/s41390-025-03986-2 PMC12875882 40128590

[2] J. Wang , C. Dai , H. Yuan , et al., “The YAP/SCAP/SREBP1 Pathway in Astrocytes: A Novel Target for Treating Neonatal Hypoxic‐Ischemic Encephalopathy,” Progress in Neurobiology 257 (2025): 102869. 10.1016/j.pneurobio.2025.102869 41390137

[3] L. Wu , E. Chang , H. Zhao , and D. Ma , “Regulated Cell Death in Hypoxic‐Ischaemic Encephalopathy: Recent Development and Mechanistic Overview,” Cell Death Discov 10, no. 1 (2024): 277. 10.1038/s41420-024-02014-2 PMC11167026 38862503

[4] Y. Yang , Y. Li , W. Yang , et al., “Protecting Effects of 4‐Octyl Itaconate on Neonatal Hypoxic‐Ischemic Encephalopathy via Nrf2 Pathway in Astrocytes,” Journal of Neuroinflammation 21, no. 1 (2024): 132. 10.1186/s12974-024-03121-8 PMC11102208 38760862

[5] M. K. Jones , A. Nair , and M. Gupta , “Mast Cells in Neurodegenerative Disease,” Frontiers in Cellular Neuroscience 13 (2019): 171. 10.3389/fncel.2019.00171 PMC6524694 31133804

[6] Z. Rubíková , V. Sulimenko , T. Paulenda , and P. Dráber , “Mast Cell Activation and Microtubule Organization Are Modulated by Miltefosine Through Protein Kinase C Inhibition,” Frontiers in Immunology 9 (2018): 1563. 10.3389/fimmu.2018.01563 PMC6046399 30038620

[7] A. E. Russi , M. E. Walker‐Caulfield , Y. Guo , C. F. Lucchinetti , and M. A. Brown , “Meningeal Mast Cell‐T Cell Crosstalk Regulates T Cell Encephalitogenicity,” Journal of Autoimmunity 73 (2016): 100–110. 10.1016/j.jaut.2016.06.015 PMC6364701 27396526

[8] U. Ocak , P. Eser Ocak , L. Huang , et al., “Inhibition of Mast Cell Tryptase Attenuates Neuroinflammation via PAR‐2/p38/NFκB Pathway Following Asphyxial Cardiac Arrest in Rats,” Journal of Neuroinflammation 17, no. 1 (2020): 144. 10.1186/s12974-020-01808-2 PMC7199326 32366312

[9] L. Priego‐González , P. P. Pardo , and F. Redegeld , “The Role of Mast Cells in Autism Spectrum Disorder,” Neuroscience and Biobehavioral Reviews 176 (2025): 106263. 10.1016/j.neubiorev.2025.106263 40523550

[10] T. C. Theoharides , S. Asadi , and A. B. Patel , “Focal Brain Inflammation and Autism,” Journal of Neuroinflammation 10 (2013): 46. 10.1186/1742-2094-10-46 PMC3626551 23570274

[11] C. C. J. Lin , F. Herisson , H. Le , et al., “Mast Cell Deficiency Improves Cognition and Enhances Disease‐Associated Microglia in 5XFAD Mice,” Cell Reports 42, no. 9 (2023): 113141. 10.1016/j.celrep.2023.113141 PMC10634538 37713312

[12] D. Kempuraj , G. P. Selvakumar , R. Thangavel , et al., “Glia Maturation Factor and Mast Cell‐Dependent Expression of Inflammatory Mediators and Proteinase Activated Receptor‐2 in Neuroinflammation,” Journal of Alzheimer's Disease 66, no. 3 (2018): 1117–1129. 10.3233/JAD-180786 PMC6334644 30372685

[13] Y. Jin , A. J. Silverman , and S. J. Vannucci , “Mast Cells Are Early Responders After Hypoxia‐Ischemia in Immature Rat Brain,” Stroke 40, no. 9 (2009): 3107–3112. 10.1161/STROKEAHA.109.549691 19520991

[14] A. C. Blanchard , A. Maximova , T. Phillips‐Jones , et al., “Mast Cells Proliferate in the Peri‐Hippocampal Space During Early Development and Modulate Local and Peripheral Immune Cells,” Developmental Cell 60, no. 6 (2025): 853–870.e7. 10.1016/j.devcel.2024.11.015 PMC11945645 39662467

[15] B. Qin , Y. Peng , C. Zhong , et al., “Mast Cells Mediate Inflammatory Injury and Aggravate Neurological Impairment in Experimental Subarachnoid Hemorrhage Through Microglial PAR‐2 Pathway,” Frontiers in Cellular Neuroscience 15 (2021): 710481. 10.3389/fncel.2021.710481 PMC8503547 34646122

[16] L. Rajendran and R. C. Paolicelli , “Microglia‐Mediated Synapse Loss in Alzheimer's Disease,” Journal of Neuroscience 38, no. 12 (2018): 2911–2919. 10.1523/JNEUROSCI.1136-17.2017 PMC6596066 29563239

[17] S. Hong , V. F. Beja‐Glasser , B. M. Nfonoyim , et al., “Complement and Microglia Mediate Early Synapse Loss in Alzheimer Mouse Models,” Science 352, no. 6286 (2016): 712–716. 10.1126/science.aad8373 PMC5094372 27033548

[18] M. W. Salter and B. Stevens , “Microglia Emerge as Central Players in Brain Disease,” Nature Medicine 23, no. 9 (2017): 1018–1027. 10.1038/nm.4397 28886007

[19] T. Faust , G. Gunner , and D. P. Schafer , “Mechanisms Governing Activity‐Dependent Synaptic Pruning in the Mammalian CNS,” Nature Reviews Neuroscience 22, no. 11 (2021): 657–673. 10.1038/s41583-021-00507-y PMC8541743 34545240

[20] J. Wu , J. Zhang , X. Chen , et al., “Microglial Over‐Pruning of Synapses During Development in Autism‐Associated SCN2A‐Deficient Mice and Human Cerebral Organoids,” Molecular Psychiatry 29, no. 8 (2024): 2424–2437. 10.1038/s41380-024-02518-4 38499656

[21] Y. Hu , H. Qi , J. Yang , et al., “Wogonin Mitigates Microglia‐Mediated Synaptic Over‐Pruning and Cognitive Impairment Following Epilepsy,” Phytomedicine 135 (2024): 156222. 10.1016/j.phymed.2024.156222 39547095

[22] S. Zhang , X. Zeng , H. Yang , G. Hu , and S. He , “Mast Cell Tryptase Induces Microglia Activation via Protease‐Activated Receptor 2 Signaling,” Cellular Physiology and Biochemistry 29, no. 5–6 (2012): 931–940. 10.1159/000171029 22613992

[23] S. Lakatos and J. Rosta , “Bidirectional Interplay Between Microglia and Mast Cells,” International Journal of Molecular Sciences 26, no. 15 (2025): 7556. 10.3390/ijms26157556 PMC12347062 40806683

[24] Y. Liu , H. Li , J. Hu , et al., “Differential Expression and Distinct Roles of Proteinase‐Activated Receptor 2 in Microglia and Neurons in Neonatal Mouse Brain After Hypoxia‐Ischemic Injury,” Molecular Neurobiology 59, no. 1 (2022): 717–730. 10.1007/s12035-021-02594-5 34762231

[25] J. Wei , A. Chen , D. Huang , et al., “Gut Microbiome‐Derived Lipopolysaccharides Aggravate Cognitive Impairment via TLR4‐Mediated Inflammatory Signaling in Neonatal Rats Following Hypoxic‐Ischemic Brain Damage,” Brain, Behavior, and Immunity 127 (2025): 4–24. 10.1016/j.bbi.2025.02.029 40010549

[26] R. Lin , Y. Zhou , T. Yan , et al., “Directed Evolution of Adeno‐Associated Virus for Efficient Gene Delivery to Microglia,” Nature Methods 19, no. 8 (2022): 976–985. 10.1038/s41592-022-01547-7 35879607

[27] A. Huo , J. Wang , Q. Li , et al., “Molecular Mechanisms Underlying Microglial Sensing and Phagocytosis in Synaptic Pruning,” Neural Regeneration Research 19, no. 6 (2024): 1284–1290. 10.4103/1673-5374.385854 PMC11467947 37905877

[28] R. C. Paolicelli , G. Bolasco , F. Pagani , et al., “Synaptic Pruning by Microglia Is Necessary for Normal Brain Development,” Science 333, no. 6048 (2011): 1456–1458. 10.1126/science.1202529 21778362

[29] U. Ocak , P. E. Ocak , A. Wang , et al., “Targeting Mast Cell as a Neuroprotective Strategy,” Brain Injury 33, no. 6 (2019): 723–733. 10.1080/02699052.2018.1556807 30554528

[30] H. Dong , X. Zhang , Y. Wang , X. Zhou , Y. Qian , and S. Zhang , “Suppression of Brain Mast Cells Degranulation Inhibits Microglial Activation and Central Nervous System Inflammation,” Molecular Neurobiology 54, no. 2 (2017): 997–1007. 10.1007/s12035-016-9720-x 26797518

[31] X. Zhang , W. Wu , Z. Zheng , et al., “Mast Cell Stabilizer Disodium Cromoglycate Improves Long‐Term Cognitive Impairment After General Anesthesia Exposure in Neonatal Mice,” Frontiers in Neuroscience 16 (2022): 990333. 10.3389/fnins.2022.990333 PMC9521828 36188474

[32] A. M. Wynne , C. J. Henry , Y. Huang , A. Cleland , and J. P. Godbout , “Protracted Downregulation of CX3CR1 on Microglia of Aged Mice After Lipopolysaccharide Challenge,” Brain, Behavior, and Immunity 24, no. 7 (2010): 1190–1201. 10.1016/j.bbi.2010.05.011 PMC2939290 20570721

[33] P. Henrich‐Noack , M. Riek‐Burchardt , K. Baldauf , G. Reiser , and K. G. Reymann , “Focal Ischemia Induces Expression of Protease‐Activated receptor1 (PAR1) and PAR3 on Microglia and Enhances PAR4 Labeling in the Penumbra,” Brain Research 1070, no. 1 (2006): 232–241. 10.1016/j.brainres.2005.10.100 16403464

[34] S. Quarta , M. Sandre , M. Ruvoletto , et al., “Inhibition of Protease‐Activated Receptor‐2 Activation in Parkinson's Disease Using 1‐Piperidin Propionic Acid,” Biomedicine 12, no. 7 (2024): 1623. 10.3390/biomedicines12071623 PMC11274518 39062196

[35] Y. Liu , Z. Wu , Y. Nakanishi , et al., “Infection of Microglia With *Porphyromonas gingivalis* Promotes Cell Migration and an Inflammatory Response Through the Gingipain‐Mediated Activation of Protease‐Activated Receptor‐2 in Mice,” Scientific Reports 7, no. 1 (2017): 11759. 10.1038/s41598-017-12173-1 PMC5603557 28924232

[36] X. Zeng , S. Zhang , L. Xu , H. Yang , and S. He , “Activation of Protease‐Activated Receptor 2‐Mediated Signaling by Mast Cell Tryptase Modulates Cytokine Production in Primary Cultured Astrocytes,” Mediators of Inflammation 2013 (2013): 140812. 10.1155/2013/140812 PMC3684029 23818741

