# Supplementary material for: Mast Cell–Microglia Crosstalk Drives Aberrant Synaptic Pruning Through PAR‐2/MAPK/NF‐κB Signaling in Neonatal Hypoxic–Ischemic Encephalopathy
Source: CNS Neurosci Ther. 2026 Sep 28;32(10):e71189. doi: 10.1002/cns.71189 (PMC13620283; doi:10.1002/cns.71189)
Supplement: Supplementary file 1 [file CNS-32-e71189-s001.docx]

**Supplementary Materials and Methods**

**1.1 Animals and experimental design**

All animal procedures adhered to the Guide for the Care and Use of Laboratory Animals. Pregnant specific-pathogen-free Sprague-Dawley rats (E13; Fujian Medical University, China) were housed under controlled temperature and humidity with a 12-h light/dark cycle and ad libitum access to food and water. On postnatal day 7（P7）, pups weighing 14-18 g were eligible for the study; pups outside this prespecified range were excluded before group allocation. Eligible pups of both sexes were assigned within litters using a computer-generated random-number sequence, with allocation balanced across litters and sex as far as practicable. To minimize observer bias, behavioral assessments, image acquisition and quantification, and Western blot densitometric analyses were performed by investigators blinded to group allocation. For statistical analyses, datasets were coded and group identities were revealed only after completion of the prespecified analyses. Histological, immunofluorescence, Western blot, molecular, and synaptic analyses used four independent animals per group, whereas behavioral assessments used eight animals per group. Sample sizes were determined based on our preliminary experimental results, the stable reproducibility of the weight-matched P7 hypoxic-ischemic (HI) model, and previously published neonatal HI studies with consistent experimental endpoints^1–3^, while limiting animal use in accordance with the 3R principles. The neonatal HI injury model was established in P7 rat pups via the classic Rice-Vannucci method, as described previously^4^.

**1.2 Histological staining and 2,3,5-triphenyltetrazolium chloride (TTC) staining**

For histological evaluation, brain tissues were fixed in 4% paraformaldehyde, dehydrated through a graded ethanol series, cleared in xylene, embedded in paraffin, and serially sectioned coronally at an appropriate thickness. After deparaffinization and rehydration, hematoxylin and eosin (H&E) staining was performed to assess the general tissue morphology and cellular architecture, whereas Nissl staining was used to evaluate neuronal integrity and the extent of neuronal loss. Apoptotic cell death was detected using a terminal deoxynucleotidyl transferase-mediated dUTP nick-end labeling (TUNEL) assay according to the manufacturer’s instructions, with nuclei counterstained with DAPI. TUNEL-positive cells were quantified within predefined regions of interest and expressed as the percentage of total DAPI-positive nuclei. For infarct assessment, freshly collected coronal brain slices were subjected to TTC staining, and the infarct area was quantified based on the absence of red staining.

**1.3 RNA sequencing and library construction**

Hippocampal tissues were collected from P8 Sham and hypoxia–ischemia (HI) rat pups. Sham animals underwent a neck skin incision without carotid artery ligation, whereas HI animals were subjected to the established HI procedure. Approximately 25 mg of hippocampal tissue from each sample was rapidly collected, snap-frozen in liquid nitrogen, and stored at −80°C until RNA extraction. Total RNA was isolated using TRIzol reagent according to the manufacturer’s instructions. RNA concentration and purity were initially assessed using a NanoDrop spectrophotometer, and RNA integrity was evaluated by agarose gel electrophoresis. For transcriptome library preparation, mRNA was enriched using the NEBNext mRNA enrichment system. The enriched mRNA was subsequently fragmented and reverse-transcribed into first-strand cDNA, followed by second-strand cDNA synthesis. Strand-specific sequencing libraries were prepared using the KAPA library preparation kit, including end repair, adapter ligation, amplification, and purification steps. The concentration and size distribution of the resulting libraries were evaluated using an Agilent Bioanalyzer, and the expected library size was approximately 400–600 bp. Qualified libraries were subjected to high-throughput paired-end sequencing on an Illumina NovaSeq 6000 platform by Gene Denovo Biotechnology Co., Ltd. Raw sequencing data were subsequently processed using standard quality-control and bioinformatic procedures for downstream transcriptomic analysis.

**1.4 Behavioral Assessments of Acute Neurological Impairment**

Behavioral tests were performed 24 h after HI modeling in neonatal rats to evaluate acute neurological deficits. All tests were conducted according to our previous methods and published protocols5. Each pup was tested individually using the righting reflex, cliff avoidance, and negative geotaxis paradigms. In the righting reflex test, the pup was placed in a supine position, and the latency to return to the normal prone position was recorded. The cliff avoidance test evaluated the pup’s ability to detect and withdraw from the edge of an elevated platform, with the latency to avoid the cliff recorded. For the negative geotaxis test, the pup was placed head-down on an inclined surface, and the time required to rotate 180° and orient toward the upper side of the slope was measured. Animals that failed to complete the required response within the predefined observation period were recorded as having an impaired response according to the experimental criteria. The recorded latencies and response outcomes were used for comparative evaluation of acute neurological function after HI injury.

**1.5 Intranasal drug administration**

FSLLRY-NH2 (F-NH2; HY-P1260, MedChemExpress, China) was used as a PAR-2 antagonist, whereas APC366 (HY-105999B, MedChemExpress, China) was used as a tryptase inhibitor. Both compounds were dissolved in 20% ethanol and administered intranasally as described previously^6–8^. Briefly, rats were placed in a supine position under 3% isoflurane anesthesia. APC366 (100 μg/kg) or F-NH2 (100 μg/kg) was administered 1 h after HI modeling, whereas vehicle-treated animals underwent the same procedure with 20% ethanol. A total volume of 30 μL was delivered over 10 min, with 5 μL administered into alternating nares every 2 min. To determine whether F‑NH₂ or APC366 modulated basal PAR‑2/MAPK/NF‑κB signaling, as well as the expression of synaptic‑ and microglia‑related proteins in the absence of HI injury, Sham+Veh, Sham+F‑NH₂, and Sham+APC366 groups were included for supplementary analyses. These sham control groups received identical drug dosages and intranasal dosing regimens as their corresponding HI‑treated counterparts.

**1.6 Microglia-targeted F2rl1 silencing**

On postnatal day 1, rat pups were anesthetized with 2%–3% isoflurane and fixed in a neonatal stereotaxic frame. AAV9-CX3CR1-shF2rl1 or AAV9-CX3CR1-shNC was bilaterally injected into the dorsal hippocampal CA1 region (AP −1.6 mm, ML ±3.0 mm, and DV −1.8 mm from bregma and the skull surface). Viral suspension (0.5 μL per side) was infused at 50 nL/min, and the needle was retained for 10 min before withdrawal. After recovery, the pups were returned to the dam. HI or sham surgery was subsequently performed on P7, and hippocampal tissues were collected on P8. Viral spread and F2rl1 knockdown efficiency were validated by reporter fluorescence imaging and quantitative real‑time PCR, respectively.^9,10^

**1.7 BV-2 cell OGD/R and pharmacological treatments**

BV-2 microglial cells were maintained in high-glucose Dulbecco’s modified Eagle’s medium supplemented with 10% fetal bovine serum and 1% penicillin–streptomycin at 37°C in a humidified atmosphere containing 5% CO₂. At 70%–80% confluence, the cells were washed twice with phosphate-buffered saline, transferred to glucose-free DMEM, and incubated in a hypoxic chamber containing 95% N₂ and 5% CO₂ for 2 h. Reoxygenation was initiated by replacing the glucose-free medium with complete culture medium and returning the cells to normoxic conditions for 24 h. Cells were randomized into five experimental groups: Ctrl+Veh, OGD/R+Veh, OGD/R+F‑NH₂, OGD/R+tryptase, and OGD/R+tryptase+F‑NH₂. Tryptase (1 μg/mL) was administered at the onset of reoxygenation. For PAR‑2 inhibition, cells were pretreated with F‑NH₂ (200 μM) for 1 h prior to tryptase stimulation, and F‑NH₂ was kept in the culture medium throughout the reoxygenation period. Corresponding control groups received an equal volume of vehicle solvent. At the end of reoxygenation, cells were harvested for immunofluorescence staining and Western blot analysis targeting CD68, PAR‑2, p38 MAPK, p‑p38 MAPK, NF‑κB p65, and p‑NF‑κB p65.

**1.8 Western blotting**

Protein expression was analyzed by Western blotting according to previously describe procedures^11^. Total proteins were extracted from the hippocampus and BV2 microglia, and protein concentrations were determined prior to electrophoresis. Equal amounts of protein were separated by SDS–PAGE, transferred onto polyvinylidene difluoride membranes, and incubated with the appropriate primary antibodies, followed by horseradish peroxidase-conjugated secondary antibodies. Total p38 MAPK and total NF-κB p65 were quantified relative to β-actin, whereas phosphorylated proteins were normalized to their corresponding total proteins (p-p38 MAPK/p38 MAPK and p-NF-κB p65/NF-κB p65). Detailed information for primary and secondary antibodies used in Western blotting, including their sources, catalog numbers, and working dilutions, is summarized in Table S1

**1.9 Immunofluorescence staining**

Paraffin-embedded coronal brain sections were deparaffinized twice in xylene for 10 min and rehydrated through 100%, 95%, 80%, and 70% ethanol. Antigen retrieval was performed by microwaving the sections in 0.01 M citrate buffer (pH 6.0) for 15 min. After cooling, endogenous peroxidase activity was blocked with 3% H₂O₂ for 10 min. The sections were washed in TBS containing 0.001% Triton X-100 and blocked with TBS containing 0.5% Triton X-100 and 10% normal horse serum for 1 h at room temperature.

The first primary antibody was diluted in blocking buffer and incubated overnight at 4°C. After washing twice in TBS-Triton for 5 min, the sections were incubated with an appropriate HRP-conjugated secondary antibody for 1 h at room temperature, followed by the assigned tyramide fluorophore at 1:500 for 10 min. For subsequent staining with a primary antibody raised in the same species, the sections were boiled in citrate buffer for 15 min to remove the primary antibody–secondary antibody–HRP complex while retaining the covalently deposited tyramide signal. After cooling and washing, the sections were blocked again, and the staining cycle was repeated with the next primary antibody and a spectrally distinct tyramide fluorophore. After the final staining cycle, nuclei were counterstained with DAPI for 10 min, and the sections were mounted with antifade medium. Primary antibody omission, secondary-only, single-marker, and post-stripping controls were included. Images were acquired using sequential confocal scanning under identical settings. Quantification was performed on the original single-channel images; pseudocolors were assigned only for figure presentation. Detailed information regarding primary antibodies, staining sequence, TSA fluorophores, and assigned pseudocolors, as well as the vendor sources and working dilutions of all primary and secondary antibodies, is summarized in Table S1.

**Table S1. Antibodies used in this study.**

| **Antibody** | **Host species/isotype** | **Vendor (City, State, catalogue)** |
| --- | --- | --- |
| anti-tryptase | rabbit | 1:2000 (WB), 1:250（IF）, ab151757, Abcam, USA |
| PAR-2 | rabbit | 1:1000 (WB), 1:100（IF）,, ab180953, Abcam, USA |
| p38 MAPK | rabbit | 1:2000, #9212, CST, USA |
| p-p38 MAPK | rabbit | 1:1000, 28796-1-AP, proteintech, CHN |
| NF-κB p65 | rabbit | 1:1000, ab16502, Abcam, USA |
| p-NF-κB p65 | rabbit | 1:1000 (WB), #3033, CST, USA |
| C3 | rabbit | 1:200（IF）, 21337-1-AP, proteintech, CHN |
| CD68 | rabbit | 1:2000 (WB), 1:200（IF）, ab283654, Abcam, USA |
| PSD95 | rabbit | 1:2000 (WB), 1:100（IF）, ab238135, Abcam, USA |
| c-Kit | rabbit | 1:100（IF）, #3074, CST, USA |
| Iba1 | rabbit | 1:200（IF）, ab178846, Abcam, USA |
| Neun | rabbit | 1:300（IF）, ab177487, Abcam, USA |
| GFAP | rabbit | 1:300（IF）, ab68482, Abcam, USA |
| C1q | rabbit | 1:200（IF）, YM960510, Immunoway, CHN |
| CX3CR1 | rabbit | 1:100（IF）, YT5112, Immunoway, CHN |
| SYP | rabbit | 1:2000 (WB), 1:100（IF）, ab128274, Abcam, USA |
| β-actin | rabbit | 1:5000, 66009-1-Ig, proteintech, CHN |
| goat-anti-rabbit secondary | rabbit | 1:25000, ab6721, Abcam, USA |

**1.10 Transmission electron microscopy**

Fresh hippocampal tissues were carefully dissected and immediately immersed in an immunoelectron microscopy fixative at 4°C. Following fixation, the samples were thoroughly rinsed with buffer, dehydrated through a graded ethanol series, infiltrated with resin, and polymerized to obtain solid tissue blocks. Ultrathin sections approximately 70–80 nm thick were cut using an ultramicrotome and collected on appropriate electron microscopy grids. For immunolabeling, the sections were blocked with 1% bovine serum albumin (BSA) to reduce nonspecific binding and incubated overnight at 4°C with the corresponding primary antibody. After extensive washing, the sections were incubated with a gold-conjugated secondary antibody at room temperature. The grids were subsequently washed, counterstained with uranyl acetate, and examined using a transmission electron microscope. Immunopositive structures were identified by the presence of electron-dense 10-nm colloidal gold particles, and the distribution of immunogold labeling was evaluated in the selected hippocampal regions.

**1.11 Golgi staining**

Whole brains were rapidly removed and immersed in Golgi fixative according to the manufacturer’s instructions. The tissues were maintained in the Golgi solution for 14 days to allow complete impregnation and were protected from excessive light during this procedure. Following impregnation, the brains were sectioned coronally at a thickness of 60 μm. The sections were then developed using the reagents provided in the Golgi staining kit, dehydrated through graded ethanol, cleared, and mounted with coverslips. Golgi-impregnated neurons were visualized under a bright-field microscope, and images were acquired from the hippocampal regions of interest. Neurons with clearly defined somata and dendritic processes were selected for morphological analysis. Dendritic morphology, including the integrity and branching characteristics of dendritic processes, was assessed using CaseViewer software according to predefined analysis criteria.

**1.12 Behavioral tests of cognitive function**

Behavioral assessments of cognitive function were initiated at postnatal day 30 (P30), corresponding to 23 days after HI injury. The Y-maze and Morris water maze tests were conducted according to established protocols, with minor modifications based on our previous studies^1,5^. The Y-maze was used to evaluate spontaneous alternation behavior and spatial working memory. Each rat was allowed to explore the three-arm maze freely, and the sequence and number of arm entries were recorded. Spontaneous alternation was calculated based on successive entries into all three arms without repeated entry into the same arm, and the alternation percentage was used as an indicator of working-memory performance. The Morris water maze was used to assess spatial learning and reference memory. During the acquisition phase, animals were trained to locate a hidden platform using spatial cues surrounding the maze. Escape latency, swimming path length, and swimming speed were recorded during each trial. A probe trial was subsequently performed in the absence of the platform to assess spatial memory retention, based on the time spent in the target quadrant and the number of crossings over the former platform location. All behavioral data were collected and analyzed using the corresponding behavioral tracking system.

**1.13 Statistical analysis**

Statistical analyses were performed using SPSS 22.0 software (SPSS Inc., USA). Normality was assessed using the Shapiro-Wilk test, and homogeneity of variance was evaluated using Levene's test. Normally distributed data are presented as mean ± standard error of the mean (SEM). For in vivo experiments, n denotes the number of independent animals; for BV-2 experiments, n denotes independently performed culture experiments. Measurements obtained from multiple tissue sections, microscopic fields, cells, or technical replicates from the same animal or culture experiment were averaged to generate one value for statistical analysis. The exact n for each experiment is provided in the corresponding figure legend. Two-group comparisons were performed using two-tailed unpaired Student's t-tests. Multiple groups were compared using one-way ANOVA followed by Bonferroni post hoc tests. Repeated-measures two-way ANOVA followed by Bonferroni post hoc tests was used for group-by-time comparisons. P < 0.05 was considered statistically significant.

**Reference**

1.	Deng J, Liao Y, Chen J, et al. N6-methyladenosine demethylase FTO regulates synaptic and cognitive impairment by destabilizing PTEN mRNA in hypoxic-ischemic neonatal rats. *Cell Death Dis*. 2023;14(12):820.

2.	Chen T, Hu Y, Lu L, et al. Myricetin attenuates hypoxic-ischemic brain damage in neonatal rats via NRF2 signaling pathway. *Frontiers in Pharmacology*. 2023;14:1134464.

3.	Lin Q, Hu DW, Hao XH, Yang H, Zeng L, Chen X. Effect of hypoxia-ischemia on the expression of iron-related proteins in neonatal rat brains. *Neural Plasticity*. 2023;2023:4226139.

4.	Chen X, Chen A, Wei J, et al. Dexmedetomidine alleviates cognitive impairment by promoting hippocampal neurogenesis via BDNF/TrkB/CREB signaling pathway in hypoxic-ischemic neonatal rats. *CNS Neurosci Ther*. 2024;30(1):e14486.

5.	Yang Y, Li Y, Yang W, et al. Protecting effects of 4-octyl itaconate on neonatal hypoxic-ischemic encephalopathy via Nrf2 pathway in astrocytes. *J Neuroinflammation*. 2024;21(1):132.

6.	Ocak U, Eser Ocak P, Huang L, et al. Inhibition of mast cell tryptase attenuates neuroinflammation via PAR-2/p38/NFκB pathway following asphyxial cardiac arrest in rats. *J Neuroinflammation*. 2020;17(1):144.

7.	Ocak U, Ocak PE, Huang L, et al. Inhibition of PAR-2 Attenuates Neuroinflammation and Improves Short-Term Neurocognitive Functions Via ERK1/2 Signaling Following Asphyxia-Induced Cardiac Arrest in Rats. *Shock*. 2020;54(4):539-547.

8.	Ocak U, Eser Ocak P, Huang L, Zhang JH. FSLLRY-NH2 Improves Neurological Outcome After Cardiac Arrest in Rats. *Turk Neurosurg*. 2020;30(2):244-251.

9.	Wei J, Chen A, Huang D, et al. Gut microbiome-derived lipopolysaccharides aggravate cognitive impairment via TLR4-mediated inflammatory signaling in neonatal rats following hypoxic-ischemic brain damage. *Brain Behav Immun*. 2025;127:4-24.

10.	Lin R, Zhou Y, Yan T, et al. Directed evolution of adeno-associated virus for efficient gene delivery to microglia. *Nature Methods*. 2022;19(8):976-985.

11.	Qian H, Chen A, Lin D, et al. Activation of the CD200/CD200R1 axis improves cognitive impairment by enhancing hippocampal neurogenesis via suppression of M1 microglial polarization and neuroinflammation in hypoxic-ischemic neonatal rats. *Int Immunopharmacol*. 2024;128:111532.
