# Supplementary material for: Mast Cell–Microglia Crosstalk Drives Aberrant Synaptic Pruning Through PAR‐2/MAPK/NF‐κB Signaling in Neonatal Hypoxic–Ischemic Encephalopathy
Source: CNS Neurosci Ther. 2026 Sep 28;32(10):e71189. doi: 10.1002/cns.71189 (PMC13620283; doi:10.1002/cns.71189)
Supplement: Supplementary file 2 [file CNS-32-e71189-s002.docx]

**Supplementary figure 1**

**Supplementary fig. 1. Establishment and characterization of the neonatal HI rat model.** (A, B) Representative TTC-stained coronal sections showing infarcted (white) regions in the ipsilateral hemisphere of HI rats compared with sham controls. Quantification reveals that infarction volume accounts for approximately 30% of the hippocampal region. (C, D) Nissl staining of hippocampal CA1 regions indicates a marked reduction in surviving neurons following HI compared with shams. (E, F) Representative TUNEL staining images demonstrate increased apoptotic cell death in hippocampal tissues from HI rats relative to sham controls. Quantification of TUNEL-positive cells confirms significant elevation of apoptosis post-HI. n = 4 animals per group. Statistical significance was assessed using two-tailed Student’s t-test or one-way ANOVA with appropriate post hoc tests. Significance levels are shown in the graphs. Scale bars=50 μm.

**Supplementary figure 2**

**Supplementary fig. 2. Increased mast cells and tryptase correlate with acute neurological impairment in HI mice.** (A-H) Correlation analysis between MCs counts (x-axis) and individual behavioral scores (y-axis): A) righting time, B) cliff-avoidance time, C) geotaxis latency, D) suspension time. (E–H) Simple linear regressions between relative tryptase expression (x-axis) and the same behavioral parameters: E) righting time, F) cliff-avoidance time, G) geotaxis latency, H) suspension time. Shaded areas indicate 95% confidence intervals; regression equations and coefficients of determination (R²) are displayed on each graph.

**Supplementary figure 3**

**Supplementary Fig. 3. F-NH2 attenuates HI-associated PAR-2 signaling and microglial synaptic engulfment.** (A) Representative immunofluorescence images showing Iba1 (green), PAR-2 (red), and DAPI (blue) in the hippocampus, together with enlarged views and single-channel images. Scale bars=100 μm (B-D) Quantification of Iba1^+^/PAR-2^+^ cells, corresponding fluorescence-intensity surface plots of PAR-2, and PAR-2 fluorescence intensity, respectively. (E) Representative immunoblots of PAR-2, p-p38 MAPK, p38 MAPK, p-NF-κB p65, NF-κB p65, and β-actin in hippocampal tissue. (F, I, J) Quantification of PAR-2, p38 MAPK, and NF-κB p65 normalized to β-actin. (G, H) Quantification of p-p38 MAPK/p38 MAPK and p-NF-κB p65/NF-κB p65 ratios, respectively. (K) Representative immunoblots of CD68, PSD95, SYP and β-actin. (L–N) Quantification of CD68, PSD95 and SYP expression normalized to β-actin, respectively. (O) Representative confocal images of Iba1^+^ microglia (red) and PSD95 (green), together with merged images and 3D reconstructions. Scale bars=10 μm (P, Q) Quantification of PSD95 puncta engulfed by Iba1^+^ microglia and PSD95 volume within Iba1-positive microglia, respectively. n = 4 animals per group. Data are presented as mean ± SEM, *p < 0.05, **p < 0.01, ***p < 0.001. Statistical significance was assessed using one-way ANOVA with appropriate post hoc tests. Significance levels are shown in the graphs.

**Supplementary figure 4**

**Supplementary Fig. 4. Sham treatment with F-NH2 or APC366 does not significantly alter PAR-2/MAPK/NF-κB signaling or synaptic and microglia-associated protein expression.** (A) Representative immunoblots of PAR-2, p-p38 MAPK, p38 MAPK, p-NF-κB p65, NF-κB p65, CD68, PSD95, SYP and β-actin in hippocampal tissue. (B) Quantification of the p-p38 MAPK/p38 MAPK ratio. (C) Quantification of the p-NF-κB p65/NF-κB p65 ratio. (D) Quantification of PAR-2, p38 MAPK, NF-κB p65, CD68, PSD95 and SYP normalized to β-actin. n = 4 animals per group. Data are presented as mean ± SEM, *p < 0.05, **p < 0.01, ***p < 0.001. Statistical significance was assessed using one-way ANOVA with appropriate post hoc tests. Significance levels are shown in the graphs.

**Supplementary figure 5**

**Supplementary fig. 5: Exogenous tryptase potentiates OGD/R-associated CD68 expression and p38 MAPK/NF-κB phosphorylation in BV-2 cells, which are attenuated by F-NH2.** (A) Representative immunofluorescence images of BV-2 cells stained for Iba1 (green), CD68 (red), and DAPI (blue), together with merged images and fluorescence-intensity surface plots under the indicated conditions: Ctrl+Veh, OGD/R+Veh, OGD/R+F-NH2, OGD/R+tryptase, and OGD/R+tryptase+F-NH2. Scale bars=10 μm. (B) Representative immunoblots of CD68, p-p38 MAPK, p-NF-κB p65 in BV-2 cells exposed to the indicated conditions. (C) Quantification of PAR-2 expression normalized to β-actin. (D) Quantification of the p-p38 MAPK/p38 MAPK ratio. (E) Quantification of the p-NF-κB p65/NF-κB p65 ratio. (F) Quantification of CD68 expression normalized to β-actin. (G) Quantification of total p38 MAPK expression normalized to β-actin. (H) Quantification of total NF-κB p65 expression normalized to β-actin. n = 4 per group. Data are presented as mean ± SEM, *p < 0.05, **p < 0.01, ***p < 0.001. Statistical significance was assessed using one-way ANOVA with appropriate post hoc tests. Significance levels are shown in the graphs.
